# Guidelines for the Selection of Scintillators for Indirect Photon-Counting X-ray Detectors

**DOI:** 10.1021/acs.chemmater.4c03437

**Published:** 2025-02-26

**Authors:** J. Jasper van Blaaderen, Casper van Aarle, David Leibold, Pieter Dorenbos, Dennis R. Schaart

**Affiliations:** † Delft University of Technology, Faculty of Applied Sciences, Department of Radiation Science and Technology, Mekelweg 15, 2629 JB Delft, The Netherlands; ‡ Holland Proton Therapy Center, Huismansingel 4, 2629 JH Delft, The Netherlands

## Abstract

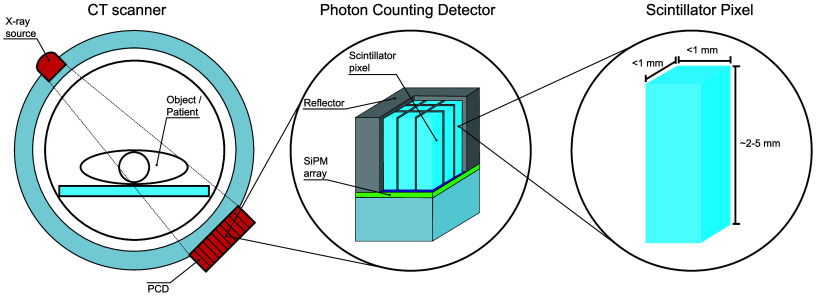

X-ray photon-counting detectors (PCDs) are a rapidly developing technology. Current PCDs used in medical imaging are based on CdTe, CZT, or Si semiconductor detectors, which directly convert X-ray photons into electrical pulses. An alternative approach is to combine ultrafast scintillators with silicon photomultipliers (SiPMs). Here, an overview is presented of different classes of scintillators, with the aim of assessing their potential application in scintillator-SiPM based indirect X-ray PCDs. To this end, three figures of merit (FOMs) are defined: the pulse intensity, the pulse duration, and the pulse quality. These FOMs quantify how characteristics such as light yield, pulse shape, and energy resolution affect the suitability of scintillators for application in indirect PCDs. These FOMs are based on emissive characteristics; a fourth FOM (ρZ_*eff*_^3.5^) is used to also take stopping power into account. Other important properties for the selection process include low self-absorption, low after-glow, possibility to produce sub-mm pitch pixel arrays, and cost-effectiveness. It is shown that material classes with promising emission properties are Ce^3+^- or Pr^3+^-doped materials, near band gap exciton emitters, plastics, and core–valence materials. Possible shortcomings of each of these groups, e.g., suboptimal emission wavelength, nonproportionality, and density, are discussed. Additionally, the engineering approach of quenching the scintillator emission, resulting in a targeted shortening of the decay time, and the possibility of codoping are explored. When selecting and/or engineering a material, it is important to consider not only the characteristics of the scintillator but also relevant SiPM properties, such as recharge time and photodetection efficiency.

## I Introduction

X-ray computed tomography (CT) is a widely used medical imaging technique. Most CT scanners today use a pixelated energy-integrating detector (EID), which yields a signal proportional to the total energy deposited in each pixel during the exposure time. As a result, no discrimination is made between the energy of incident X-rays, with high-energy X-rays contributing more to the signal. Moreover, electronic noise is integrated during the exposure time. EIDs are thus limited in signal-to-noise and contrast-to-noise ratio for a given radiation dose.^1,2^

Photon-counting detectors (PCDs) can help mitigate these problems. A PCD counts the number of incident X-ray photons hitting the detector during the exposure time, only registering a count when the electrical output pulse of the PCD passes a predefined threshold. As a result, most of the electronic noise is rejected. In the case of a purely counting detector, all X-ray photons contribute equally to the detector signal. The use of multiple thresholds makes it possible to assign counts to different energy bins, thus yielding an energy-resolving PCD, which enables the use of PCDs in dual- or multienergy (spectral) X-ray imaging.

Despite their advantages, PCDs are thus far not widely implemented in medical X-ray imaging systems. One of the challenges to their implementation is the high X-ray fluence rates that occur in medical imaging. Due to the detector’s finite response time, multiple X-ray photons hitting a pixel within this response time result in a superposition of their respective electrical output pulses. This is termed pulse pile-up. It can lead to the registration of a count in an energy bin with an energy higher than the incident X-ray photon’s energy. It has been estimated by Persson et al. that the maximum fluence rate reached in a standard clinical CT protocol is approximately 3.4 × 10^8^ photons/s/mm^2^; however, the fluence rate in most regions of a patient’s shadow on the detector are much lower than the maximum.^3^ In order to be able to handle this fluence rate and to reduce pulse pile-up, the detector pulse duration should not exceed more than a few tens of nanoseconds. Additionally, miniaturization of the pixels, to a size smaller than 0.5 × 0.5 mm^2^, helps to distribute the incident photons over multiple pixels, reducing the rate requirement per pixel. This does, however, increase the probability that a characteristic or Compton scattered X-ray photon escapes from a pixel and is absorbed in a neighboring pixel. This is referred to as interpixel X-ray crosstalk and increases pulse pile-up and reduces spatial resolution.^4^ Further requirements for PCDs are a sufficient full width at half-maximum (fwhm) energy resolution of the detector system in the diagnostic energy range (25 to 150 keV), sufficient density to achieve high X-ray stopping power, and room temperature operation.

The potential of PCDs for CT has been explored extensively in the last 10 years.^1,5−8^ Current PCDs are based on direct detection, converting the energy deposited by X-ray photons into a charge pulse using semiconductors such as CdTe,^9^ Cd_1–*x*_Zn_*x*_Te (CZT),^10^ or Si.^11^ Several photon-counting CT systems have been constructed and are being used to evaluate their benefits in clinical practice.^1,12^ These systems can reach energy resolutions in the range of 10 to 20% at 60 keV.^10,13^ Nevertheless, CdTe- and CZT-based PCDs face several challenges, for example, the production of detector-grade semiconductors with sufficiently low defect density,^1,2^ which can affect cost-effectiveness. Another challenge is the occurrence of charge sharing between pixels,^10^ which leads to errors in both the number of registered counts and their energies.^14^ Additionally the low hole mobility of CdTe and CZT can give result in polarization effects at high count rates.^1^ Additionally, the effects of long-term exposure to the high X-ray fluence rates on detector performance of CdTe and CZT-based detectors is yet to be investigated.^5^ For Si-based detectors, the low atomic number (*Z* = 14) and density (ρ = 2.3 g/cm^3^) makes Compton scattering the dominant interaction mechanism of X-ray photons^4^ within the pixels, degrading both spectral performance and spatial resolution.^15^

**Figure 1:**
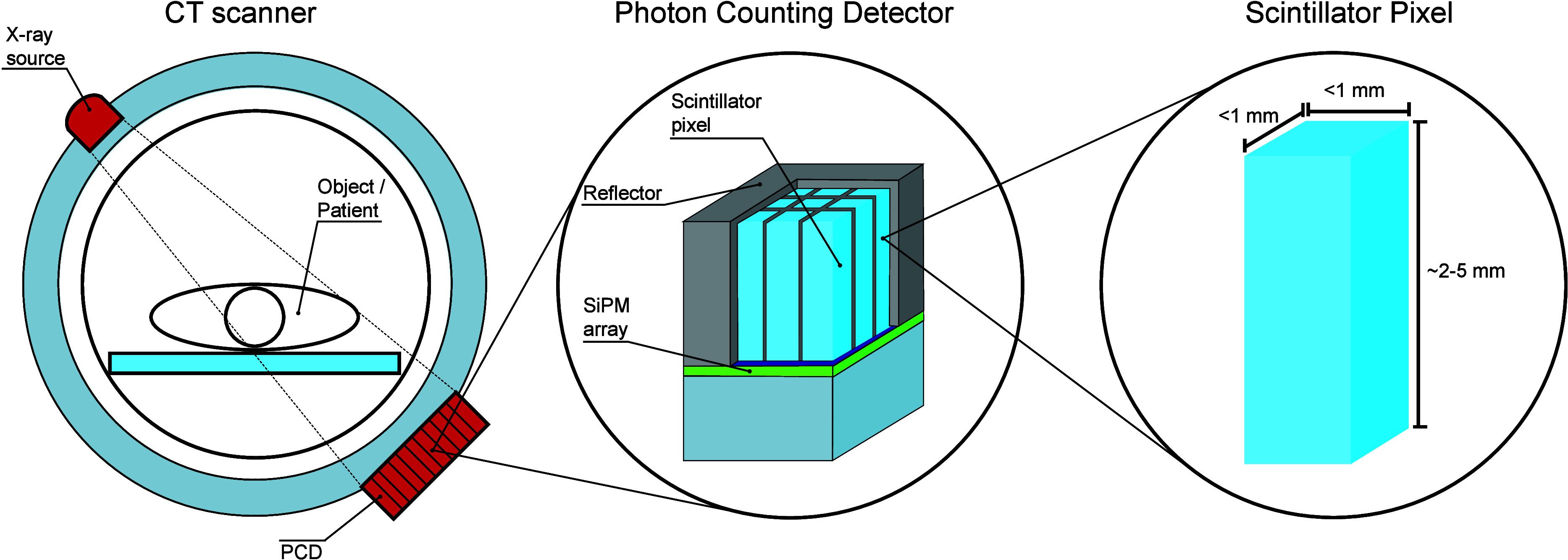
Schematic overview of, from left to right, a simplified photon-counting CT set up, a simplified overview of the pixilated PCD for which part of the reflector is removed to show the scintillator pixels, and one of the scintillator pixels with the approximate crystal size.

An alternative to the above-mentioned direct PCDs are indirect detectors, utilizing ultrafast scintillators to absorb the incident X-rays and convert them into scintillation photons. Scintillators have proven to be reliable and cost-effective detector materials in a plethora of medical imaging systems, including positron emission tomography (PET), single-photon emission tomography (SPECT), and energy-integrating CT systems.^16−21^ Moreover, scintillators do not suffer from polarization or charge sharing effects. Therefore, indirect PCDs may perform better in spectral imaging tasks.^14^ The scintillation photons are detected and converted into an electrical pulse using a photodetector. A simplified schematic of a scintillator based, or indirect, PCD is shown in Figure 1. From left to right, it shows a simplified photon-counting CT set up, a pixelated PCD, and a single scintillator pixel crystal. In an indirect PCD, the scintillator pixels are separated and covered on the outside by reflectors in order to efficiently collect the scintillation photons and avoid optical crosstalk between pixels. Part of the outside reflector is removed in the diagram to show the scintillator pixels underneath. In this work, the focus will be on the use of silicon photomultipliers (SiPMs) as the photodetectors, due to their internal gain, short response time, compactness, robustness, and the possibility to implement sub-mm pixel sizes.^22−25^ A SiPM pixel consists of a two-dimensional (2D) array of single-photon avalanche diodes (SPADs) with a size on the order of 10 μm. When a photon hits a SPAD, an avalanche of charge carriers is created. Since the SPADs within a SiPM are connected in parallel, the output signal is proportional to the number of scintillation photons incident on the SiPM, provided that the number of SPADs is sufficiently large compared to the number of scintillation photons. The SiPM should have a short recharge time and a photodetection efficiency curve matching the emission spectrum of the scintillator.

In an indirect PCD, the scintillator should have a sufficiently high density to efficiently absorb the incident X-rays and minimize X-ray cross-talk between pixels. The scintillator should also have a short decay time in order to handle high X-ray fluence rates. Initial feasibility studies on SiPM-based scintillator PCDs have been performed by Van der Sar et al. by coupling (Lu_1–*x*_Y_*x*_)_2_SiO_5_:Ce^3+^ (LYSO:Ce^3+^), YAlO_3_:Ce^3+^ (YAP:Ce^3+^), LuAlO_3_:Ce^3+^ (LAP:Ce^3+^), and LaBr_3_:Ce^3+^ to a single-pixel SiPM with a recharge time of 7 ns.^22−25^ The pulse duration, defined as the fwhm of the detector output pulse, of the LaBr_3_:Ce^3+^ based detector was experimentally determined to be 26.5 ns fwhm.^23^ Using the same definition, the pulse duration of a typical CdTe-based detector is 14 ns fwhm.^14^ Additionally, a fwhm energy resolution of 8% at 60 kV has been demonstrated for CdTe-based PCDs.^10^ Experimentally, the LaBr_3_:Ce^3+^-based detector showed a fwhm energy resolution of 20% at 60 keV.^22,24,25^ In those studies, however, the energy resolution was limited by the low (<25%) photodetection efficiency of the used SiPM. It has been demonstrated by Alekhin et al. that LaBr_3_:Ce^3+^ can reach an energy resolution of 9.4% at 60 keV.^26^

Another study, by Arimoto et al., used a 1 × 64 array of 1 × 1 mm^2^ pixels coupled to a cerium-doped yttrium–gadolinium–aluminum–gallium garnet (YGAGG) scintillator.^27^ Arimoto et al. demonstrated that, with an energy resolution of 32% at 60 keV, it was possible to acquire energy-resolved X-ray images and perform material decomposition.^28,29^ However, the X-ray fluence rate was restricted by the 70 ns decay time of the YGAGG scintillator. Sato et al. also used YGAGG to construct a table-top preclinical CT system, demonstrating accurate density maps of gadolinium and iodine.^30^ Another example is the work of Shimazoe et al., who build a pixelated detector based on gadolinium–aluminum–gallium garnet (GAGG)/gadolinium–fine aluminum–gallate (GFAG) scintillator pixels coupled to a finely pixelated SiPM array.^31^ The detector consisted of a 10 × 10 scintillator pixel array of 200 μm^2^ separated by a optical reflection layer with a thickness of 50 μm. The scintillators used in this detector, (GAGG)/(GFAG), have decay times of more than 50 ns, restricting the range of fluence rates under which the detector can operate.

In this work, an overview of different types of scintillators is presented. The goal is to identify suitable materials for use in indirect PCDs. To this end, three different figures of merit (FOMs) are formulated: the pulse intensity, the pulse duration, and the pulse quality. These FOMs aim to quantify how scintillator emissive characteristics such as light yield, pulse shape, and energy resolution affect the suitability of the material for application in indirect PCDs. Additionally, a fourth FOM is used to assess the X-ray stopping power of the material. Next to the presented material selection, potential challenges for different classes of scintillators are identified. The presented framework is also used to explore the possibility of engineering a scintillator for use in an indirect PCD.

## II Signal Formation in an Indirect PCD

In order to assess how the emission characteristics of different scintillators influence the performance of an indirect PCD, a simple model has been formulated. The model describes how the essential parts of the detection chain affect the signal formation, taking into account properties of both the scintillation crystal and the SiPM. The model can be subdivided into four different stages, from the generation of X-ray photons to the electrical output pulse of the indirect PCD.

### II.A Stage I

In the first stage, an X-ray photon is created and passes through the object, e.g., a patient. After this, the X-ray photon with energy *E*_X_ impinged on a pixel of the detector. It then either interacts with the scintillator or passes through without depositing any energy. The probability of interaction is determined by the density and elemental composition of the scintillation material, as well as the pixel dimensions.

In case the X-ray photon interacts, it deposits an amount of energy *E*_dep_ in the scintillator-pixel. *E*_dep_ is not always the same as *E*_X_ and depends on the probability of secondary photons, i.e., Compton scattered photons, or characteristic X-rays, escaping the pixel. These probabilities again depend on the material density and composition, as well as the dimensions of the pixel and the location of interaction of the X-ray photon within it.

### II.B Stage II

In the second stage, the energy deposited by the interacting X-ray is converted to a flash of scintillation photons. The average number of emitted scintillations photons for a given amount of deposited energy is given by
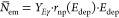
1Here, *Y*_*Eγ*_ represents the light yield of the scintillator per unit of energy for a given amount of deposited energy, often 662 keV from γ-photons of ^137^Cs. Ideally, the light yield would be independent of the amount of deposited energy. In reality, however, this is not the case. The relative variation of the light yield with deposited energy is called nonproportionality and is represented by the term *r*_np_(*E*_dep_). A more elaborate discussion of the role of nonproportionality is provided in Section III.A.

### II.C Stage III

In the third stage, the scintillation photons are transported through the crystal toward the SiPM where each photon may trigger a SPAD, in which case it is detected. The expected number of detected scintillaton photons (*N*_det_) is given by
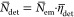
2Here, η_det_ represents the effective photon detection efficiency (PDE) of the detector. The effective PDE is defined as
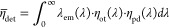
3Here, λ_em_(λ) represents the emission spectrum of the scintillator, normalized according to
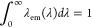
4while η_ot_(λ) is the optical transfer efficiency of the detector (taking into account e.g. the optical properties of the scintillator and optical coupling material, the reflectivity of the reflectors, etc.), and η_pd_(λ) is the photon detection efficiency of the SiPM. The effective PDE thus depends on properties of the scintillator, the photodetector, and any other relevant components of the detector.

### II.D Stage IV

In the fourth and final stage, the SiPM generates an electrical pulse. In reality, the charge *Q*(*E*_X_) contained in this pulse depends not only on the number of detected scintillation photons but also on the nonproportional response of the SiPM due to saturation effects, afterpulsing, and crosstalk. The role of these effects on the response of a SiPM-based scintillation detector has been discussed comprehensively by van Dam et al.^32^ In a well-designed detector, one attempts to mitigate these effects so as to obtain a response that is as proportional as reasonably achievable. Moreover, the acquired detector signals can be corrected for any remaining nonproportionality, for example using the model by van Dam et al.^32^ For simplicity, we will therefore assume that *Q*(*E*_X_) is proportional to *N*_det_ in the remainder of this work.

The electrical output pulse of the detector can be described by convolution of the single SPAD response and the decay profile of the scintillation pulse. The response of a single fired SPAD can be approximated as an exponentially decaying current:
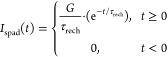
5where *G* and τ_rech_ are the gain and the recharge time constant of the SiPM, respectively.

The expected time distribution of the emitted scintillation photons, on the other hand, can be described by
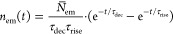
6Here, τ_rise_ and τ_dec_ are the rise and decay time constants of the scintillator, respectively.

Due to the submillimeter size of the scintillator crystal, it can be assumed that the optical transfer times of the scintillation photons can be neglected. The time distribution of the number of triggered SPADs, i.e., the number of detected scintillation photons, can therefore be described to a good approximation by
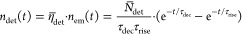
7If the rise time is significantly shorter than the decay time, this result can be further simplified to
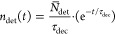
8In reality, a measured scintillator decay curve may show nonexponential behavior or consist of multiple decay components. In order to still arrive at a simple model, such decay behavior can be approximated using a single exponential. In this work, a weighted average is used for the decay time constant in the case of multiple decay components. This is discussed more elaborately in Section IV and eq 16.

Following the above discussion, the time evolution of the detector output pulse can be calculated by convoluting eqs 5 and 8:
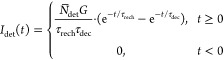
9

## III Figures of Merit

Based on the above discussion, three different FOMs can be formulated to quantify the performance potential of a scintillator in indirect PCDs. The FOMs are the pulse intensity, the pulse duration, and the pulse quality.

### III.A Pulse Intensity

The pulse intensity is simply defined as the expected number of detected scintillation photons, *N*_det_, defined in eq 2. As discussed in Section II.C, it depends on both *N*_em_ and η_det_ making it a property related to the scintillator, SiPM, and any other relevant components of the detector.

*N*_det_ is closely related to the energy resolution *R* of the detector. To explain this, we can make use of a formulation of the energy resolution of a scintillation detector that is frequently used in literature:^33−36^
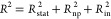
10Here, *R*_stat_^2^ represents the statistical variance in the number of photons detected by the photon detector and *R*_np_^2^ the variance due to the nonproportionality of the scintillator. The variance due to crystal inhomogeneities influencing the energy resolution, e.g., a nonuniform distribution of dopant ions and/or surface effects, is represented by *R*_in_^2^. In an indirect PCD, the influence of *R*_in_^2^ is expected to be small due to the submillimeter size of the scintillation crystals. For completion, it is noted that eq 10 does not take into account electronic contributions as the focus of this work is on scintillation materials.

Nonproportionality, *r*_np_(*E*_dep_), is a material property describing the relative variation of the light yield *Y*_Eγ_ with deposited energy, referred to as the energy *E*_γ_ at which *Y*_Eγ_ was determined.^36,37^ It has been studied extensively, both experimentally^38−41^ and theoretically^42−45^ in the last two decades. Traditionally, scintillator energy resolutions are determined using the 662 keV γ-photons of ^137^Cs.^33^ At this energy, nonproportionality tends to have a relatively large influence on the scintillator energy resolution, and consequently, the statistical limit of the energy resolution can only be reached when *r*_np_(*E*_dep_) is close to an ideal response, i.e., *r*_np_(*E*_dep_) = 1 for all values of *E*_dep_. In the diagnostic energy range (25 to 150 keV), on the other hand, the dominant factor influencing the energy resolution is *R*_stat_^2^ (which is discussed in more detail below). This means that the energy resolution improves when the nonproportionality is larger than 1 in the diagnostic energy range. On the other hand, when the nonproportionality is smaller than 1, the energy resolution will deteriorate. Experimentally, this means that characterizing a scintillator using the 59.5 keV gamma photons of ^241^Am instead of the 662 keV gamma photons of ^137^Cs will provide much more relevant information with respect to their application in indirect PCDs.

The term *R*_stat_ depends on the number of detected scintillation photons. It can be described by a binomial distribution. In practice, however, it is commonly approximated by a Poisson distribution. Hence, assuming Poisson statistics,
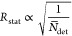
11In case *R*_in_, *R*_np_, and all other contributions to the energy resolution can be considered negligible, eq 11 defines the lower limit on the energy resolution of a scintillator-based PCD. Theoretically, the best value of *R*_stat_ is achieved for η_det_ = 1, resulting in *N*_det_ = *N*_em_ (see eq 2). This means that each of the emitted scintillation photons reaches the SiPM and triggers a SPAD. In practice, η_det_ < 1 and one attempts to maximize its value by optimizing the optical transfer efficiency of the detector and matching the PDE curve of the SiPM to the scintillator emission spectrum (see eq 3).

In summary, the energy resolution that can be achieved with an indirect PCD scales inversely proportional to the square root of the number of detected scintillation photons. Moreover, *N*_det_ should be evaluated in the diagnostic energy range, for example using 59.5 keV gamma photons of ^241^Am, in order to take the nonproportionality of the scintillator into account.

### III.B Pulse Duration

The pulse duration (*t*_fwhm_) is defined as the fwhm of the average detector output pulse *I*_det_(*t*). It is noted that we can facilitate the (numerical) calculation of the fwhm of eq 9, by first normalizing this function such that the peak value is always equal to one:^46,47^
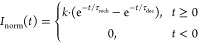
12Here, the normalization constant *k* is defined as
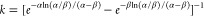
13where α and β are defined as 1/τ_rech_ and 1/τ_dec_, respectively.

**Figure 2:**
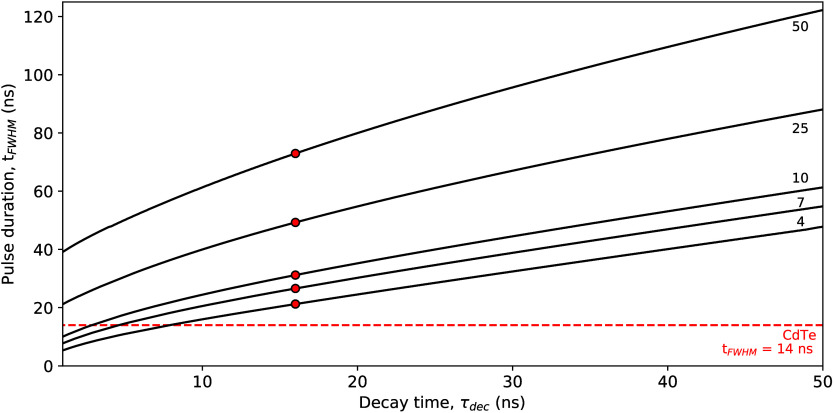
Calculated pulse duration (*t*_fwhm_) as a function of the scintillator decay time (τ_dec_) for different recharge times (τ_rech_). The red dashed line represents a state-of-the-art-pulse duration of 14 ns fwhm of a CdTe detector. The red markers indicate the calculated pulse duration of a LaBr_3_:Ce^3+^-based PCD for different recharge times.

Figure 2 shows the calculated pulse duration (*t*_fwhm_) as a function of the scintillator decay time constant, for different recharge time constants ranging from 4 to 50 ns. For reference, the pulse duration of CdTe based detector, 14 ns fwhm, is indicated by the red dashed line.^14^ Since several CdTe-based direct PCDs have been used to evaluate their use in clinical practice, it can be assumed that a pulse duration of at most 14 ns fwhm is sufficient to handle the high X-ray fluency rate of medical imaging systems.^1,12^ As a second reference, red circular markers are added at 16 ns, which represent the decay time of LaBr_3_:Ce^3+^. For a SiPM with τ_rech_ = 7 ns, as was used experimentally by van der Sar et al.,^22−25^ the calculated pulse duration of LaBr_3_:Ce^3+^ is equal to 26.5 ns fwhm. Decreasing τ_rech_ to 4 ns reduces the pulse duration to 21.2 ns fwhm. This shows that further decreasing the recharge time of the SiPM only results in marginal improvement of the pulse duration of a LaBr_3_:Ce^3+^-based PCD due to the fact that a recharge time of 7 ns is already significantly shorter than the 16 ns scintillation decay time. The relative influence of τ_rech_ will be larger for a faster scintillator.

### III.C Pulse Quality

The pulse quality (PQ) is defined as
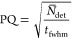
14This FOM equals the square root of the ratio of the pulse intensity and duration. In case τ_dec_ ≪ τ_rech_, the shape of *I*_det_(*t*) is mainly determined by τ_rech_ and can therefore be considered constant, see eq 9. The uncertainty in the amplitude of *I*_det_(*t*) is then determined by the Poisson variation of *N*_det_ and, therefore, inversely proportional with PQ. If, on the other hand, τ_rech_ ≪ τ_dec_, the average shape of *I*_det_(*t*) is mainly determined by τ_dec_. In that case, a smaller value of PQ implies not only a larger uncertainty in the amplitude of *I*_det_(*t*), but also an increase of the statistical fluctuations in the shape of *I*_det_(*t*), due to the random emission times of the scintillation photons. If these statistical pulse shape fluctuations become too strong, a single pulse can pass the detection threshold repeatedly and be counted multiple times. In principle, statistical pulse shape fluctuations can be smoothened out by low-pass filtering, but this may lead to unwanted pulse elongation.^22,24,25^ In summary, larger values of PQ indicate better potential for achieving accurate counting and energy binning.

### III.D Stopping Power

Another material property that is important for an initial material selection is the stopping power of the scintillator. The stopping power of a scintillator is determined by its mass density and the effective atomic number. Ideally, the stopping power of a scintillator should be high enough to absorb all of the energy of the incident X-ray photon. Moreover, photoelectric absorption should be the main mechanism of interaction between the scintillator pixel and the incident X-ray photons. The higher the effective atomic number of a scintillator, the higher the probability of photoelectric absorption taking place. In order to take the stopping power into account a fourth FOM (ρZ_*eff*_^3.5^) will be used. This product provides an indication of the stopping power and efficiency of photoelectric absorption. The larger this number, the better the stopping power and the larger the probability of photoelectric absorption. The effective attomic number (Z_*eff*_) is defined as^48^
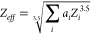
15It should be noted that the number ρZ_*eff*_^3.5^ provides only an initial indication of the stopping power. The stopping power of a scintillator for X-rays in the diagnostic energy range (25 to 150 keV) will depend on the exact composition of the scintillator and the position of the K and L-edges.

### III.E Purpose and Scope of the Proposed FOMs

The four figures of merit described above are based on the emissive properties of a scintillator combined with the detection properties of a SiPM, and the density and composition of the scintillator. A candidate material should have a high pulse intensity, a short pulse duration, a high value of pulse quality, and a high value of ρZ_*eff*_^3.5^. Such a material will efficiently absorb X-ray photons and convert them into a short and intense pulse of scintillation light, with a low probability of pulse pile-up and low statistical fluctuations of the detector output pulse amplitude and shape. It should be noted that other material properties, such as self-absorption, afterglow, radiation hardness, possibility to produce submillimeter pitch pixel arrays, and cost-effectiveness, are not taken into account in the FOMs formulated and discussed above. Such properties are of high importance for the applicability of a material in real-world PCDs. It should thus be noted that the FOMs presented here are only to be used for an initial selection of candidate materials.

## IV Scintillator Overview

An initial assessment of scintillators for their application in PCDs can be made by plotting the pulse quality over the pulse duration. However, not all scintillator and detector properties are readily available in the literature. For a first general material selection, the following assumptions are therefore made. First, the effective PDE (η_det_) is taken as 100%. Second, scintillator light yields are commonly determined and reported by using the 662 keV γ-photons of ^137^Cs. This value is used to estimate the intensity of the scintillation pulse (*N*_em_) because data on nonproportionality or the light yield at clinically relevant X-ray energies is not always available. This results in *N*_det_ = *N*_em_, which is taken to be equal to the light yield measured at 662 keV. Finally, not all scintillators show a single-exponential decay (cf. eq 8). For scintillators which show multiple decay components the average decay time constant is calculated according to
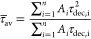
16Here, *A*_*i*_ represents the amplitude and τ_dec,i_ the decay time of the different decay components. The separate decay components can be found in the Supporting Information.

Based on the above three assumptions, data was collected from literature to asses the performance potential of different scintillators. The tabulated values of the energy resolution are defined as the fwhm of the photopeak at 662 keV. The tabulated values of the peak emission wavelength are defined as the peak wavelength of the most intense emission band. The tabulated values of the pulse duration are calculated based on a convolution of either the reported single-exponential decay time or the calculated average decay time, in combination with a SiPM recharge time constant of 7 ns. The tabulated values of the pulse quality are calculated based on the tabulated values of the light yield and pulse duration.

Solid-state scintillators can be subdivided into two general groups: extrinsically activated scintillators and intrinsic scintillators. Extrinsically activated scintillators can be divided into subgroups based on the used activator ion; typical activator ions are Ce^3+^, Eu^2+^, and Tl^+^. In the past three decades, scintillation research has mainly focused on the development of Ce^3+^ and Eu^2+^-doped compounds. An example is the development of LaBr_3_:Ce^3+^, which has a light yield of 76 ph/keV and decay time of 16 ns.^26^ Alekhin et al. showed that codoping LaBr_3_:Ce^3+^ with Sr^2+^ improves the proportionality without affecting the light yield. This resulted in the current record energy resolution of 2% at 662 keV. Co-doping with Sr^2+^, however, introduces several slow decay processes. Due to the additional weight that is given to slower decay components (eq 16), this results in an average decay time of 1.2 μs for LaBr_3_:Ce^3+^,Sr^2+^.^26,49^ Other well-known Ce^3+^ based scintillators are Lu_2_SiO_5_:Ce^3+^^50,51^ and (Lu,Y)_2_SiO_5_:Ce^3+^^52−54^ which are used in positron emission tomography scanners due to their high density and relatively short decay time of around 40 ns.^55^ Not all Ce^3+^-doped scintillators show Ce^3+^ 5d → 4f emission exclusively. Some also show emission originating from the host matrix. An example of this is Cs_2_LiYCl_6_:Ce^3+^, commonly used for the detection of neutrons.^56−58^ The host emission introduces a slow component in the scintillation decay. The collected data on Ce^3+^-doped scintillators are summarized in Table I.

Examples of Eu^2+^-doped scintillators are SrI_2_Eu^2+^^59−61^ and CsBa_2_I_5_:Eu^2+^^62,63^ with light yields of 90 and 97 photons/keV, respectively. The Eu^2+^ 4f^6^5d → 4f^7^ transition, however, has a typical decay time between 500 and 1000 ns, making these scintillators 10–50 times slower than Ce^3+^-doped scintillators. Additionally, Eu^2+^-doped scintillators often suffer from self-absorption, due to a relatively small Stokes shift.^63−68^ This is mainly a problem in applications where crystals larger than several millimeters are required. The collected data on Eu^2+^-doped scintillators are summarized in Table II.

Two other divalent lanthanides that have been used as activator ions are Yb^2+^ and Sm^2+^. Yb^2+^ is very similar to Eu^2+^ in spin-allowed decay time and emission wavelength. However, its decay always includes a slow millisecond component due to the spin-forbidden 4f^13^5d → 4f^14^ transition.^69^ Sm^2+^, on the other hand, emits in the near-infrared and has a decay time of approximately 2 μs.^70^ It can be used as a codopant next to Eu^2+^ or Yb^2+^, as their energy can efficiently be transferred to Sm^2+^.^71−75^ The collected data on Yb^2+^, Eu^2+^-Sm^2+^, and Yb^2+^-Sm^2+^ are summarized in Table III.

Another, less commonly used, lanthanide activator ion is Pr^3+^.^76−81^ It can show 4f^1^5d → 4f^2^ emission in compounds where the energy of its 4f^1^5d state lies between that of the 4f^2^[^1^S_0_] and 4f^2^[^3^P_2_] states.^82^ One of the main challenges of utilizing the fast 4f^1^5d → 4f^2^ transition of Pr^3+^ is that it is located in the UV.^83^ However, its shorter emission wavelength compared to the Ce^3+^ 5d → 4f emission also results in shorter decay times of typically 10 to 20 ns.^83^ This is observed in Lu_3_Al_5_O_12_:Pr^3+^, for example.^84,85^ The collected data on Pr^3+^-doped scintillators is summarized in Table III.

The Tl^+^-doped scintillators CsI:Tl^+^^86^ and NaI:Tl^+^^87,88^ have been studied extensively and are commercially available. The latter, discovered by Hofstadter in 1948,^89−91^ is one of the first and most widely used scintillators to this day. The collected data on Tl^+^-doped scintillators are summarized in Table III.

Intrinsic scintillators can be divided into four subgroups based on their emission: broad-band, near band gap excitonic, core–valence luminescence, and plastics. Each of these groups will be discussed in the next paragraphs, starting with the broad-band emitters.

Two well-known intrinsic broad-band scintillators are Bi_4_Ge_3_O_12_ (BGO) and PbWO_4_. BGO, due to its 300 ns decay time and density of 7.13 g/cm^3^, is used in positron emission tomography detectors (PET).^92−94^ PbWO_4_ has a much lower light yield, but, due to is decay time of 30 ns and density of 8.28 g/cm^3^, has been used in the Large Hadron Collider of CERN.^95,96^ Intrinsic broad-band scintillators that have been explored more recently are the cesium hafmium-based halides,^97−100^ cesium copper-based iodides,^101−103^ and thallium-based halides.^104−107^ These compounds typically show decay times on the order of 1 to 2 μs. The collected data on intrinsic broad-band emitting scintillators are summarized in Table IV.

The second subgroup is scintillators showing near band gap excitonic emission. Examples of materials in this subgroup are the inorganic perovskites,^108−112^ and the hybrid organic–inorganic perovskites and perovskite-related compounds.^113−121^ Specifically, two-dimensional perovskites have shown to be promising scintillators due to their stable room-temperature near band gap excitonic emission.^122,123^ Their nanosecond decay time makes them very interesting for use in a PCD. This possibility has been explored with benzylamonium lead bromide ((BZA)_2_PbBr_4_).^124^ Xia et al. have demonstrated that the decay time of 2D hybrid organic–inorganic perovskites can be reduced even further by engineering the dielectric constant of the organic molecule.^125,126^ A downside to these materials, however, is their low density of approximately 2.5 g/cm^3^. The collected data on the near band gap exciton scintillators are summarized in Table IV.

The third subgroup consists of scintillators showing core–valence luminescence. Such an emission originates from the recombination of an electron in the valence band with a hole in the highest core band. This is referred to as core–valence luminescence (CVL), cross luminescence, or auger-free luminescence.^127^ It can only take place when the condition formulated in eq 17 is satisfied.^128^
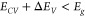
17Here, E_*CV*_ represents the energy gap between the highest core band and the bottom of the valence band, Δ*E*_*V*_ is the width of the valence band, and E_*g*_ is the band gap. This condition is satisfied in some fluorides, chlorides, and bromides containing Ba^2+^, Cs^+^, Rb^+^, and K^+^.^129^ One of the first compounds in which CVL was discovered is BaF_2_.^130^ The emission spectrum of BaF_2_ contains three CVL bands, at 183, 196, and 220 nm, in addition to the self-trapped exciton band at 310 nm.^131^ The CVL bands have a decay time of 0.8 ns, while the self-trapped exciton band has a decay time of 600 ns. The short decay time of the CVL emission makes these materials potential candidates for use in a PCD. One of the main problems, however, is the presence of STE or other emissions with longer decay times. The self-trapped exciton emission of BaF_2_ can be suppressed by doping BaF_2_ with, for example, La^3+^,^132,133^ Cd^3+^,^132,133^ Y^3+^,^134,135^ and Sc^3+^.^136^ The CVL bands of fluoride-based compounds are approximately between 140 and 230 nm. The emission wavelength shifts to longer wavelengths in chloride- and bromide-based compounds. Examples of chloride-based compounds that show CVL emission are the families of Cs–Mg–Cl and Cs–Zn–Cl compounds.^137−139^ An advantage of these compounds is the absence of slow decay components at room temperature. The collected data on fluoride- and chloride-based compounds that show CVL emission are summarized in Table V. Bromide-based compounds like CsBr, CsCaBr_3_, and CsSrBr_3_ are not considered due to their low CVL intensity.^127,129,140,141^

The last subgroup consists of plastic scintillators. These materials are often used for the detection of neutrons.^142−144^ Plastic scintillators typically have a density of about 1 g/cm^3^ and contain low Z atoms, like carbon and hydrogen, which makes these materials less suitable for the detection of high-energy γ-photons. High-Z dopants can be added to a plastic scintillator to increase its absorption.^145−147^ The collected data on plastic scintillators are summarized in Table VI.

**Table I:** Light Yield (photons/keV) Measured at 662 keV, fwhm Energy Resolution (E%) Measured at 662 keV, Decay Time Constant (τ_dec_ (ns)), Peak Emission Wavelength (λ_em_ (nm)), Mass Density (g/cm^3^), ρZ_*eff*_^3.5^ Calculated Based on Tabulated Compound Composition and Mass Density, Pulse Duration Calculated Assuming a SiPM Recharge Time Constant of 7 ns (*t_fwhm_* (ns)), and Pulse Quality Calculated Based on Tabulated Values of the Light Yield and Pulse Duration (PQ (photons/keV/ns)^1/2^) of Ce^3+^-Doped Scintillators

Compound	Light yield	E% @662 keV	τ_dec_	λ_em_	Density	ρZ_*eff*_^3.5^	*t*_fwhm_	PQ	ref
	(Ph/keV)		(ns)	(nm)	(g/cm^3^)	(10^6^)	(ns)	(Ph/keV/ns)^1/2^	
CeF_3_	4	20%	27	340	6.16	6.41	36.3	0.332	(190)
LaCl_3_	46	3.1%	169^a^	337	3.86	2.68	143	0.566	(191−193)
LuCl_3_	5.4	11.4%	5,572^a^	374/400	4	7.53	3,920	0.037	(192,194)
K_2_LaCl_5_	29	5.1%	68	380	2.89	1.47	68.6	0.650	(195)
K_2_CeCl_5_	30	5.8%	71	370	2.95	1.60	70.9	0.650	(196,197)
KGd_2_Cl_7_	30	10%	179^a^	400	3.56	3.94	151	0.446	(198)
CsSrCl_3_	8.6	7.2%	603^a^	360/385	2.96	1.77	453	0.138	(199)
Cs_3_LaCl_6_	16	8.6%	1,026^a^	380/407	3.36	3.28	750	0.146	(200)
CsCe_2_Cl_7_	28	5.5%	50	410	3.6	3.19	54.8	0.715	(201)
Cs_3_CeCl_6_	19	8.4%	265^a^	385	3.4	3.19	213	0.299	(202)
Cs_3_GdCl_6_	24	4.5%	2,008^a^	380/405	3.56	3.83	1,437	0.131	(203)
Tl_2_LaCl_5_	70	3.4%	895^a^	383	5.2	15.44	658	0.326	(204,205)
Tl_2_GdCl_5_	53	5%	1,330^a^	389	5.1	15.70	963	0.235	(206)
Rb_2_LiCeCl_6_	23	7.9%	239^a^	370	3.1	1.55	194	0.344	(207)
Rb_2_LiGdCl_6_	18	6.8%	3,490^a^	375/420	3.23	2.28	2,471	0.087	(208)
Cs_2_LiYCl_6_	22	4.5%	6,000	376/404	3.31	2.10	4,219	0.720	(209,210)
Cs_2_LiLaCl_6_	35	3.4%	445^a^	340/420	3.3	2.78	341	0.320	(211)
Cs_2_LiCeCl_6_	22	5.5%	2,263^a^	410	3.41	2.95	1,615	0.117	(212)
Cs_2_LiGdCl_6_	20	5%	7,706^a^	380/405	3.67	3.78	5,404	0.061	(213)
Cs_2_NaLaCl_6_	26	4.4%	1,513^a^	373/405	3.26	2.68	1,091	0.156	(200)
Cs_2_NaCeCl_6_	20	8.3%	2,696^a^	410	3.25	2.74	1,917	0.102	(214)
Cs_2_NaGdCl_6_	27	4%	3,030^a^	375/403	3.52	3.54	2,151	0.112	(203,215)
Tl_2_LiYCl_6_	25	4%	777^a^	435	4.5	12.50	575	0.208	(216)
Tl_2_LiGdCl_6_	58	4.6%	868^a^	376/382	n.r.	n.r.	639	0.301	(217)
Tl_2_LiLuCl_6_	27	5.6%	1,373^a^	428	5.06	15.66	993	0.165	(218)
BaBr_2_	10	9.8%	1,935^a^	375/410	4.84	3.60	1,386	0.086	(219)
LaBr_3_	76	2.7%	16	358	5.29	3.56	26.5	1.515	(26,220,221)
LaBr_3_:Ce,Sr	78	2%	1,209^a^	356/382	5.29	3.56	878	0.298	(26,49,162)
LaBr_2.25_I_0.75_	45	4.1%	117^a^	400/434	5.47	4.19	149	0.548	(222)
LaBr_1.5_I_1.5_	58	14.6%	28	472/500	5.51	4.98	37.1	1.249	(222)
LaBr_0.75_I_2.25_	22	35.9%	12	460/510	5.6	5.72	22.6	0.986	(222)
CeBr_3_	60	3.6%	17	370	5.2	3.68	27.5	1.477	(223,224)
CeBr_3_:Sr	55	3%	65^a^	381	5.2	3.68	66.3	0.911	(225−227)
PrBr_3_:20%Ce	21	6.9%	7.9	365/395	5.33	3.69	18.2	1.075	(155)
PrBr_3_:5%Ce	16	5.5%	5.6	365/395	5.33	3.69	15.3	1.022	(155)
GdBr_3_	44	10%	698^a^	419	4.6	4.53	520	0.291	(228)
LuBr_3_	32	6%	3,433^a^	408/448	5.17	7.34	2,430	0.115	(192,194)
K_2_LaBr_5_	40	5%	50	359/391	3.9	1.88	54.8	0.854	(229)
Rb_2_CeBr_5_	34	6.9%	56.1	390	4.26	2.17	59.5	0.756	(230)
RbGd_2_Br_7_	54	3.8%	60	425	4.7	4.06	62.5	0.929	(231)
Cs_3_LaBr_6_	32	4.9%	2,042^a^	395/425	4.55	3.61	1,461	0.149	(200,232)
CsCe_2_Br_7_	33	7%	1,222^a^	450	4	2.97	888	0.200	(233)
Cs_3_GdBr_6_	47	4%	1,289^a^	396/417	4.14	3.92	935	0.224	(203)
Tl_2_LaBr_5_	43	5%	25	375/315	5.9	14.02	34.6	1.115	(234)
Rb_2_LiYBr_6_	23	4.7%	1,199^a^	385/420	3.82	1.06	871	0.162	(235)
Rb_2_LiLaBr_6_	33	4.8%	978^a^	363/387	n.r.	n.r.	716	0.215	(236)
Rb_2_LiCeBr_6_	33	6.3%	155^a^	373	4.6	2.21	133	0.497	(213)
Cs_2_LiYBr_6_	23	7%	2,492^a^	389	4.15	2.38	1,775	0.115	(210,237)
Cs_2_LiLaBr_6_	60	2.9%	540^a^	380/430	4.2	3.03	408	0.383	(238−240)
Cs_2_LiCeBr_6_	28	7.4%	3,289^a^	400/418	4.18	3.08	2,330	0.110	(241)
Cs_2_LiGdBr_6_	30	7.1%	3,477^a^	392/400	4.41	3.90	2,462	0.111	(242)
Cs_2_NaYBr_6_	9.5	6.3%	2,543^a^	385/420	3.94	2.22	1,810	0.072	(243)
Cs_2_NaYBr_3_I_3_	43	3.3%	1,795^a^	425/460	n.r.	n.r.	1,288	0.183	(244)
Cs_2_NaLaBr_6_	46	3.9%	3,535^a^	382/414	3.93	2.79	2,501	0.136	(200,243)
Cs_2_NaLaBr_3_I_3_	58	2.9%	1,089^a^	430	n.r.	n.r.	794	0.270	(244)
Cs_2_NaCeBr_6_	25	6.7%	352^a^	377	4.25	3.08	275	0.301	(245)
Cs_2_NaGdBr_6_	48	3.3%	396^a^	393/422	4.18	3.63	306	0.396	(203,246)
Cs_2_ NaLuBr_6_	10	5.5%	280^a^	389/422	4.42	4.58	223	0.217	(243)
Tl_2_LiGdBr_6_	17	17%	91^a^	422	5.3	12.12	86.2	0.449	(247)
SrI_2_:Ce,Na	16	6.4%	426^a^	404/434	4.59	4.10	327	0.221	(61)
YI_3_	99	9.3%	45	532	4.62	4.38	50.8	1.395	(248)
GdI_3_	44	4.3%	45	532	5.22	7.20	50.8	0.930	(248,249)
LuI_3_	98	3.3%	85^a^	472	5.6	9.48	81.5	1.097	(250−252)
K_2_LaI_5_	55	4.5%	24	401/439	4.4	4.57	33.7	1.277	(229)
Cs_3_Lu_2_I_9_	22	9%	446^a^	429/471	4.78	7.04	342	0.258	(232)
Cs_2_NaLaI_6_	26	4.4%	1,513^a^	420/458	4.17	4.73	1,091	0.156	(200)
YAlO_3_	15	4.4%	35^a^	347	5.35	1.08	42.9	0.609	(253−255)
Y_3_Al_5_O_12_	14	12%	70	550	4.55	0.77	70.2	0.447	(256)
LuAlO_3_	11	9.3%	43^a^	365	8.34	17.62	49.3	0.481	(257)
Lu_3_Al_5_O_12_	12	12%	50	500/560	6.7	12.46	54.8	0.478	(258)
Y_2_SiO_5_	24	9.4%	42	420	4.45	1.03	48.5	0.703	(259,260)
Gd_2_SiO_5_	12	7%	336^a^	430	6.71	10.48	285	0.209	(259,261)
Gd_2_Si_2_O_7_	30	6%	46	372/394	5.5	7.53	51.6	0.762	(262)
Lu_2_SiO_5_	27	7.9%	40	420	7.4	17.06	46.9	0.759	(50)
Lu_2_SiO_5_:Ce,Ca	38	7.7%	36.7	420	7.4	17.06	44.3	0.936	(51)
Lu_2_Si_2_O_7_	26	10%	38	378	6.2	12.64	45.3	0.757	(263,264)
(Lu_0.9_Y_0.1_)_2_SiO_5_	27	8%	36	425	7.1	15.41	43.7	0.786	(52−54)
K_2_Lu(PO_4_)_2_	26	17%	1,074^a^	390	3.90	4.67	784	0.184	(265)

a Decay consists of multiple components, tabulated value was calculated using eq 16. The amplitudes and decay times of the different components can be found in Table S1.

**Table II:** Light Yield (photons/keV) Measured at 662 keV, fwhm Energy Resolution (E%) Measured at 662 keV, Decay Time Constant (τ_dec_ (ns)), Peak Emission Wavelength (λ_em_ (nm)), Mass Density (g/cm^3^), ρZ_*eff*_^3.5^ Calculated Based on Tabulated Compound Composition and Mass Density, Pulse Duration Calculated Assuming a SiPM Recharge Time Constant of 7 ns (*t*_FWHM_ (ns)), and Pulse Quality Calculated Based on Tabulated Values of the Light Yield and Pulse Duration (PQ (photons/keV/ns)^1/2^) of Eu^2+^-Doped Scintillators

Compound	Light yield	E% @662 keV	τ_dec_	λ_em_	Density	ρZ_*eff*_^3.5^	*t*_fwhm_	PQ	ref
	(Ph/keV)		(ns)	(nm)	(g/cm^3^)	(10^6^)	(ns)	(Ph/keV/ns)^1/2^	
CaF_2_	24	6.7%	900	440	3.18	0.06	662	0.190	(266,267)
BaFI	55	8.5%	500	450	5.45	6.10	380	0.380	(268,269)
BaCl_2_	52	3.5%	604^a^	402	3.89	3.40	454	0.338	(219,270)
BaClBr	52	3.55%	500	425	4.5	3.59	380	0.370	(268,269)
BaClI	54	9%	500	425	4.5	4.79	380	0.377	(268,269)
CsCaCl_3_	18	8.9%	5,050	450	3	1.80	3,558	0.071	(271)
CsSrCl_3_	33	11.5%	2,700	448	2.96	1.77	1,920	0.132	(272)
CsSrClBr_2_	35	3.6%	2,100	462	3.98	2.25	1,502	0.153	(273)
CaBr_2_	36	8.9%	2,500	448	3.35	0.70	1,780	0.142	(274)
BaBr_2_	49	6%	672^a^	408	4.78	3.56	501	0.315	(219,275)
BaBrI	91	3.4%	453^a^	413	5.18	5.09	347	0.512	(276,277)
LiSr_2_Br_5_	32	6.1%	1,418	407/476	3.76	1.04	1,024	0.177	(278)
KSr_2_Br5	75	3.5%	1,013^a^	427	3.98	1.05	741	0.318	(279)
RbCaBr_3_	43	4.0%	2,800	436	3.46	0.84	1,990	0.147	(280)
Rb_4_CaBr_6_	71	6.9%	5,360^a^	457	3.46	0.92	3,774	0.137	(280)
RbSr_2_Br_5_	64	4%	780	429	4.18	1.18	577	0.335	(281)
CsCaBr_3_	28	9.3%	5,270	447	3.68	2.02	3,710	0.087	(282)
CsCaBrI_2_	51	3.9%	3,500	445	3.59	3.26	2,478	0.145	(273)
CsCaBr_0.8_I_2.2_	40	5.2%	2,290	450	4.06	3.81	1,633	0.156	(283)
CsSrBr_3_	40	4.9%	2,300	440	3.76	2.08	1,640	0.157	(284)
CsSrBrI_2_	65	3.4%	1,800	455	4	3.53	1,292	0.225	(273)
LiI	15	7.5%	1,200	475	4.08	4.19	872	0.131	(285,286)
CaI_2_	90	5.2%	790	470	3.96	3.73	584	0.392	(287,288)
SrI_2_	90	2.6%	1,200	453	4.6	4.10	872	0.321	(59−61)
SrI_2_:Eu,Zr	95	2.5%	1,030	436	4.6	4.10	753	0.355	(163)
BaI_2_	38	5.6%	513	426	5.15	6.00	389	0.312	(275)
LiCa_2_I_5_	90	5.6%	1,416	472	4	3.83	1,023	0.297	(278)
LiSrI_3_	35	5.2%	510	420/460	n.r.	n.r.	387	0.301	(289)
LiSr_2_I_5_	60	3.5%	1,331	497	n.r.	n.r.	964	0.249	(278)
KCaI_3_	72	3%	1,060	466	3.81	3.44	774	0.305	(290)
KCaI_3_:Eu,Zr	72	2.5%	1,313^a^	450	3.81	3.44	951	0.275	(164)
KCa_0.8_Sr_0.2_I_3_	73	2.8%	1,258	475	3.81	3.42	913	0.283	(64,291)
KSr_2_I_5_	94	2.4%	2,531^a^	445	4.39	3.87	1,802	0.228	(292)
KBa_2_I_5_	90	2.4%	910	444	4.52	5.00	669	0.367	(293)
K_2_BaI_4_	63	2.9%	720	448	4.05	4.11	535	0.343	(293)
RbSrI_3_	24	2.8%	1,030	462	4.1	3.47	753	0.323	(294)
RbSr_2_I_5_	90	3%	890	445	4.55	3.93	654	0.372	(281)
CsCaI_3_	38	8%	1,720	450	4.06	4.24	1,236	0.176	(271)
Cs_4_CaI_6_	51	3.6%	1,990	459	3.99	4.44	1,425	0.191	(295,296)
CsSrI_3_	73	3.9%	3,300	452	4.25	4.29	2,338	0.177	(66)
Cs_4_SrI_6_	62	3.5%	1,430	462	4.03	4.41	1,033	0.246	(295,296)
CsBa_2_I_5_	97	2.3%	350	430	5	5.82	273	0.595	(62,63)
TlSr_2_I_5_	70	4.2%	2,465^a^	463	5.3	9.01	1,756	0.200	(297)
Cs_3_KCaI_6_	62	3.9%	1,860	472	3.94	4.20	1,334	0.216	(295)
Cs_3_KSrI_6_	29	5%	1,780	459	3.85	4.03	1,277	0.151	(295)
Cs_3_RbCaI_6_	38	4.5%	1,250	467	3.96	4.14	907	0.205	(295)
Cs_3.5_Rb_0.5_SrI_6_	75	3.3%	1,340	466	4.03	4.28	970	0.278	(295)
Cs_3_RbSrI_6_	31	5.1%	1,220	462	3.95	4.14	886	0.187	(295)

a Decay consists of multiple components, tabulated value was calculated using eq 16. The amplitudes and decay times of the different components can be found in Table S2.

**Table III:** Light Yield (photons/keV) Measured at 662 keV, fwhm Energy Resolution (E%) Measured at 662 keV, Decay Time Constant (τ_dec_ (ns)), Peak Emission Wavelength (λ_em_ (nm)), Mass Density (g/cm^3^), ρZ_*eff*_^3.5^ Calculated Based on Tabulated Compound Composition and Mass Density, Pulse Duration Calculated Assuming a SiPM Recharge Time Constant of 7 ns (*t*_FWHM_ (ns)), and Pulse Quality Calculated Based on Tabulated Values of the Light Yield and Pulse Duration (PQ (photons/keV/ns)^1/2^) of Scintillators with Activator Ions Other than Ce^3+^ or Eu^2+^

Compound	Light yield	E% @662 keV	τ_dec_	λ_em_	Density	ρZ_*eff*_^3.5^	*t*_fwhm_	PQ	ref
	(Ph/keV)		(ns)	(nm)	(g/cm^3^)	(10^6^)	(ns)	(Ph/keV/ns)^1/2^	
LaBr_3_:Pr	60	3.2%	11,000	492–682	5.29	3.56	7,695	0.088	(298)
Lu_3_Al_5_O_12_:Pr	19	4.6%	20.1	325	6.7	12.46	30.3	0.791	(84,85)
(Lu,Y)_3_Al_5_O_12_:Pr	33	4.4%	1,080^a^	325	6.2	14.04	788	0.205	(299−301)
(Lu,Y)_3_Al_5_O_12_:Pr,Li	25	4.1%	1,180^a^	325	6.2	14.04	858	0.171	(301)
SrI_2_:Eu,Sm	42	10.5%	1,500	740	4.59	4.10	1,082	0.197	(71)
CsBa_2_I_5_:Eu,Sm	45	3.2%	2,077^a^	755	5	5.82	1,485	0.174	(72)
Cs_4_EuI_6_:Sm	16	7.5%	3,500	850	4.25	5.24	2,478	0.082	(73)
CsYbI_3_:Sm	30	7%	2,300	800	4.76	7.44	1,640	0.135	(74)
NaI:Tl	38	5.4%	250	415	3.67	3.37	202	0.433	(86−88)
NaI:Tl,Ca	26	5.3%	380^a^	420	3.67	3.37	295	0.297	(302)
NaI:Tl,Sr	34	5.4%	327^a^	420	3.67	3.37	257	0.363	(302)
NaI:Tl,Ca,Eu	52	4.9%	1,000	450	3.67	3.37	732	0.266	(303,304)
CsI:Tl	54	4.8%	1,000	550	4.51	5.23	732	0.272	(86,87,305)
SrI_2_:Yb	56	4.35%	610	414	4.6	4.10	458	0.350	(306)
RbSrI_3_:Yb	24	4.9%	795	457	4.1	3.47	588	0.202	(294)
CsBa_2_I_5_:Yb	54	5.7%	870	414	5	5.82	640	0.290	(306)

a Decay consists of multiple components, tabulated value was calculated using eq 16. The amplitudes and decay times of the different components can be found in Table S3.

**Table IV:** Light Yield (photons/keV) Measured at 662 keV, fwhm Energy Resolution (E%) Measured at 662 keV, Decay Time Constant (τ_dec_ (ns)), Peak Emission Wavelength (λ_em_ (nm)), Mass Density (g/cm^3^), ρZ_*eff*_^3.5^ Calculated Based on Tabulated Compound Composition and Mass Density, Pulse Duration Calculated Assuming an SiPM Recharge Time Constant of 7 ns (*t*_FWHM_ (ns)), and Pulse Quality Calculated Based on Tabulated Values of the Light Yield and Pulse Duration (PQ (photons/keV/ns)^1/2^) of Undoped Scintillators

Compound	Light yield	E% @662 keV	τ_dec_	λ_em_	Density	ρZ_*eff*_^3.5^	*t*_fwhm_	PQ	ref
	(Ph/keV)		(ns)	(nm)	(g/cm^3^)	(10^6^)	(ns)	(Ph/keV/ns)^1/2^	
BaCl_2_	1.7	17.4%	980	300/410	3.89	3.40	718	0.049	(307)
(EDBE)PbCl_4_	9	30%	7.9	520	2.19	4.57	18.2	0.704	(121)
Cs_2_HfCl_6_	54	3.3%	2,200	375/435	3.86	5.27	1,751	0.185	(97,308)
Cs_2_ZrCl_6_	33	4.5%	1,500	440/479	3.36	2.18	1,082	0.177	(97,308)
TlMgCl_3_	30	3.7%	413^a^	409	4.43	12.96	318	0.310	(104)
TlCaCl_3_	30	5%	622^a^	425	3.77	10.54	466	0.256	(105)
Tl_2_HfCl_6_	27	3.7%	1,063^a^	460	5.1	16.10	776	0.590	(106,107)
Tl_2_ZrCl_6_	35	3.4%	2,292^a^	460	4.5	12.61	1,635	0.146	(106,107)
BaBr_2_	19	5.4%	2,200	425	4.78	3.56	1,571	0.111	(307)
(BM)_2_PbBr_4_^b^	3.2	19.53%	0.95	440	2.05	3.00	7.7	0.643	(125)
(BZA)_2_PbBr_4_^b^	3.7	8%	4.2	440	2.3	3.45	13.4	0.526	(124)
(PEA)_2_PbBr_4_^b^	11	39%	35	440	2.36	3.42	42.9	0.506	(120,309)
TlCaBr_3_	41	6.2%	2,328^a^	470	4.69	10.07	1,660	0.158	(310)
TlSr_2_Br_5_	37	4.6%	1,470^a^	441	5.03	7.35	1,061	0.188	(311)
CaI_2_	107	3.2%	834	410	3.96	3.73	616	0.417	(287,288,312)
RbSrI_3_	8	7.6%	918^a^	447	4.1	3.47	674	0.109	(294)
CsCu_2_I_3_	16	7.8%	110	560	5.01	4.64	100	0.399	(101,102)
Cs_3_Cu_2_I_5_	29	3.4%	965^a^	450	4.51	4.65	707	0.202	(103)
Cs_2_HfI_6_	64	4.2%	2,500	700	5.12	7.30	1,780	0.190	(99,100)
Cs_3_Lu_2_I_9_	6.6	19.2%	770	390/608	4.82	7.10	570	0.108	(232)
TlCaI_3_	42	6.2%	1,105^a^	460/533	4.73	10.53	805	0.229	(104)
TlSr_2_I_5_	31	8.5%	2,372^a^	463	5.3	9.01	1,691	0.135	(297)
Sc_2_O_3_	19	16.7%	290^a^	330	3.83	0.11	231	0.288	(313)
CaWO_4_	15	6.6%	8,722^a^	425	6.1	13.61	6,113	0.051	(314−316)
CdWO_4_	15	9.1%	15,000^a^	480	7.9	15.95	10,480	0.038	(266,317)
PbWO_4_	0.3	n.r.	26^a^	420	8.28	30.49	15.8	0.138	(95,318,319)
Bi_4_Ge_3_O_12_	7.6	9.05%	300	485	7.13	25.16	238	0.179	(253,261,320)

a Decay consists of multiple components, tabulated value was calculated using eq 16. The amplitudes and decay times of the different components can be found in Table S4.

b Compounds show near-bandgap exciton emission.

**Table V:** Light Yield (photons/keV) Measured at 662 keV, fwhm Energy Resolution (E%) Measured at 662 keV, Decay Time Constant (τ_dec_ (ns)), Peak Emission Wavelength (λ_em_ (nm)), Mass Density (g/cm^3^), ρZ_*eff*_^3.5^ Calculated Based on Tabulated Compound Composition and Mass Density, Pulse Duration Calculated Assuming an SiPM Recharge Time Constant of 7 ns (*t*_FWHM_ (ns)), and Pulse Quality Calculated Based on Tabulated Values of the Light Yield and Pulse Duration (PQ (photons/keV/ns)^1/2^) of Scintillators Showing Core–Valence Luminescence

Compound	Light yield	E% @662 keV	τ_dec_	λ_em_	Density	ρZ_*eff*_^3.5^	*t*_fwhm_	PQ	ref
	(Ph/keV)		(ns)	(nm)	(g/cm^3^)	(10^6^)	(ns)	(Ph/keV/ns)^1/2^	
RbF	1.7	n.r.	1.3	203/234	3.6	0.90	8.4	0.451	(321)
BaF_2_	12	11%	630^a^	220/310	4.88	4.97	472	0.159	(322−324)
CsF	1.9	20%	2	390	4.64	4.98	9.8	0.441	(325)
KMgF_3_	1.4	n.r.	1.3	140/170	3.2	0.04	8.4	0.409	(127,321,326)
KCaF_3_	1.4	n.r.	2	165/200	3	0.06	9.8	0.379	(127,321)
KYF_4_	1	n.r.	1.9	170	3.6	0.59	9.6	0.323	(127,321)
K_2_YF_5_	0.3	n.r.	1.3	170	3.6	0.48	8.4	0.189	(321)
KLuF_4_	0.2	n.r.	1.3	163/185	5.2	9.36	8.4	0.143	(127,129,321)
KLu_2_F_7_	0.3	n.r.	2	165	7.5	14.98	9.8	0.166	(321)
CsMgCl_3_	1.1	n.r.	2.36	350	3.23	2.04	10.5	0.326	(127)
CsMgCl_3_:Zn	3.4	16.7	2.17	300	3.23	2.04	10.0	0.581	(139)
Cs_2_MgCl_4_	2.2	22	2.04	300	2.95	2.26	9.8	0.473	(138,139)
Cs_2_MgCl_4_:Zn	2.4	19.2	1.91	300	2.95	2.26	9.6	0.504	(139)
Cs_3_MgCl_5_	1.3	33.7	1.46	300	3.15	2.60	8.7	0.392	(138,139)
Cs_3_MgCl_5_:Zn	2.1	22.8	1.25	300	3.15	2.60	8.3	0.514	(139)
CsCaCl_3_	1.4	n.r.	2	250/305	2.9	1.74	9.8	0.379	(321,327)
Cs_2_ZnCl_4_	2	22%	1.66	285/379	3.35	2.41	9.2	0.464	(137)
Cs_3_ZnCl_5_	1.4	25%	0.82	240/289	3.44	2.71	7.3	0.449	(137)
CsSrCl_3_	0.9	n.r.	2.07	248	2.87	1.72	10.0	0.299	(127,328)
Cs_2_BaCl_4_	1.4	n.r.	1.68	400	3.76	3.53	9.2	0.386	(127)
Cs_2_LiYCl_6_	22	11%	6,599^a^	325	3.31	2.10	4,634	0.070	(209,210)

a Decay consists of multiple components, tabulated value was calculated using eq 16. The amplitudes and decay times of the different components can be found in Table S5.

**Table VI:** Light Yield (photons/keV) Measured at 662 keV, fwhm Energy Resolution (E%) Measured at 662 keV, Decay Time Constant (τ_dec_ (ns)), Peak Emission Wavelength (λ_em_ (nm)), Mass Density (g/cm^3^), ρZ_*eff*_^3.5^ Calculated Based on Tabulated Compound Composition and Mass Density, Pulse Duration Calculated Assuming a SiPM Recharge Time Constant of 7 ns (*t*_FWHM_ (ns)), and Pulse Quality Calculated Based on Tabulated Values of the Light Yield and Pulse Duration (PQ (photons/keV/ns)^1/2^) of Plastic Scintillators

Compound	Light yield	E% @662 keV	τ_dec_	λ_em_	Density	ρZ_*eff*_^3.5^	*t*_fwhm_	PQ	ref
	(Ph/keV)		(ns)	(nm)	(g/cm^3^)	(10^6^)	(ns)	(Ph/keV/ns)^1/2^	
Anthracene	20	n.r.	31	460	1.25	-b	39.7	0.712	(329,330)
Stilbene	16	n.r.	6	390	0.97	-b	15.9	1.005	(329,331)
p-Terphenyl	19	n.r.	7.2^a^	410	1.24	-b	17.4	1.057	(329,332)
Polyvinylcarbazole:Bi	12	9%	15	420	n.r.	-b	25.6	0.684	(333)
BC-400	13	n.r.	2.4	423	1.02	-b	10.5	1.113	(334)
BC-408	13	n.r.	2.1	425	1.02	-b	10.0	1.143	(335)
BC-412	12	n.r.	3.3	434	1.02	-b	12.0	1.000	(336)
BC-428	7.2	n.r.	12.5	480	1.02	-b	23.2	0.558	(337)
BC-430	9	n.r.	16.8	580	1.02	-b	27.3	0.574	(338)
BC-452	10	n.r.	2.1	424	1.05	-b	10.0	1.003	(339)
EJ-200	10	n.r.	2.1	425	1.02	-b	10.0	1.003	(340)
EJ-240	6.3	n.r.	285	430	1.02	-b	227	0.166	(341)
EJ-260	9.2	n.r.	9.2	490	1.02	-b	19.7	0.684	(342)
EJ-256	6.8	n.r.	2.1	425	1.08	-b	10.0	0.827	(343)

a Decay consists of multiple components; the tabulated value was calculated using eq 16. The amplitudes and decay times of the different components can be found in Table S6.

b Effective atomic number could not be calculated due to lack of information on composition.

## V Scintillator Assessment

**Figure 3:**
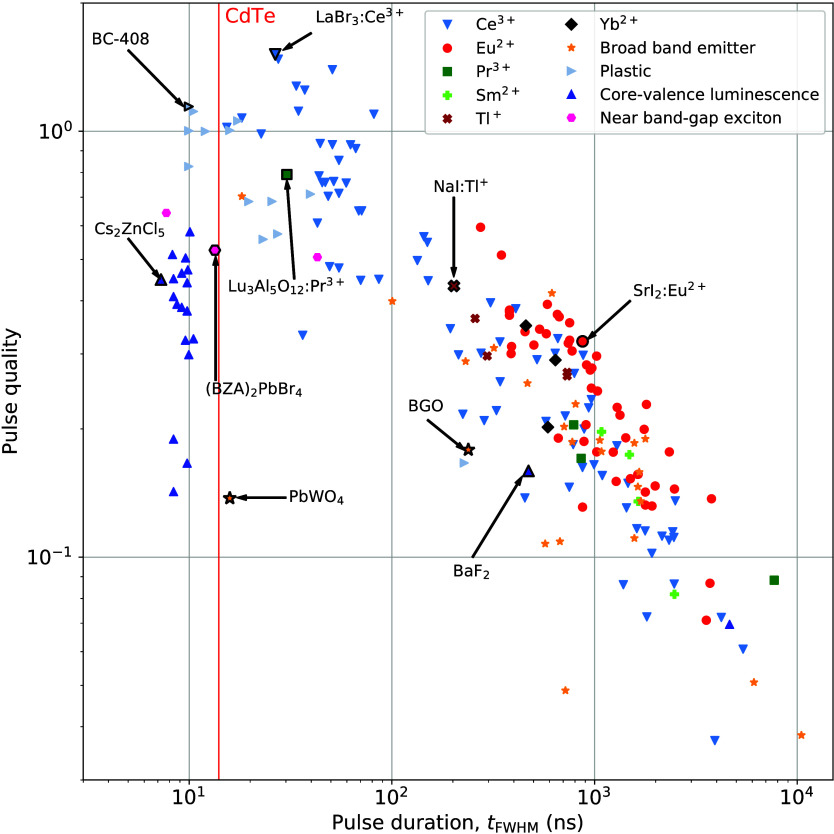
Plot of the pulse duration (*t*_fwhm_) versus the pulse quality for Ce^3+^-, Pr^3+^-, Eu^2+^-, Yb^2+^-, Sm^2+^ codoped, and Tl^+^-doped scintillators, intrinsic scintillators showing core–valence luminescence, broad-band emission, near band gap exciton emission, and plastic scintillators. The values of *t*_fwhm_ are calculated assuming a SiPM recharge time of 7 ns.

Using the FOMs and collected data discussed above, an assessment of the suitability of different scintillators for their use in indirect PCDs is made. To this end, the pulse duration is calculated as the fwhm of the average detector output pulse (eq 12), assuming a SiPM recharge time of 7 ns. The result is plotted versus the pulse quality (eq 14) in Figure 3. For reference, the pulse duration of CdTe (14 ns fwhm) is indicated by the red vertical line, providing a benchmark for the pulse duration requirements of an indirect PCD.

Figure 3 shows that most data points from Eu^2+^, Yb^2+^, Sm^2+^, Tl^+^ and broad-band emission based scintillators have pulse durations longer than 100 ns fwhm. This results in pulse qualities typically below 0.400 (photons/keV/ns)^1/2^, even though light yields of up to 90 photons/keV have been reported for compounds like SrI_2_:Eu^2+^.^59,60^ The pulse duration of Ce^3+^-doped scintillators ranges from tens of nanoseconds to tens of microseconds; the average decay time of Ce^3+^ strongly depends on the host matrix. This can be explained by inefficient or slow transfer of charge carriers toward Ce^3+^ and the presence of host-related emissions next to the intrinsic 5d → 4f emission of Ce^3+^. There are several Ce^3+^-based scintillators that approach the pulse duration of CdTe, i.e., LaBr_3_:Ce^3+^ and CeBr_3_.

The situation of Pr^3+^-doped scintillators is very similar to that of Ce^3+^-doped scintillators. The average decay time of Pr^3+^ depends strongly on the host matrix, i.e. the occurrence of efficient 5d → 4f emission. For example, Lu_3_Al_5_O_12_:Pr^3+^ shows efficient 5d → 4f emission and has a pulse duration of 30.3 ns fwhm. LaBr_3_:Pr^3+^, on the other hand, shows only 4f → 4f emission and has a pulse duration of 7.6 μs. The shorter emission wavelength of Pr^3+^ decreases its intrinsic 5d → 4f decay time compared to that of Ce^3+^. The main challenge for Pr^3+^ however is the efficient detection of its UV emission.

The shortest pulse durations are achieved by a scintillator based on CVL emission, near-bandgap exciton emission, and plastics, with some materials showing pulse durations shorter than CdTe. However, these different types of scintillators each have their respective challenges. For CVL emitters, for example, these include the presence of slow decay components or the low light yield of the CVL emission.

For near band gap exciton emitters, self-absorption is a big challenge.^114,148,149^ Self-absorption refers to the reabsorption of emitted scintillation photons within the scintillation crystal. It is a direct result of spectral overlap between the excitation and emission spectra of the scintillator; the energy difference between these two is referred to as the Stokes shift. Self-absorption can decrease the observed light yield due to a higher probability of nonradiative losses. In case the probability of re-emission after self-absorption is very high, i.e., close to 1, the influence on the observed light yield may remain small, but the observed scintillation decay may still be elongated, especially in large crystals.^150,151^ Self-absorption is also a problem in Eu^2+^-doped scintillators in which it has been studied extensively.^63−68,152,153^ It has been demonstrated that the self-absorption in Eu^2+^-doped scintillators can be mitigated by codoping these materials with Sm^2+^.^71,73,75^ The role and impact of self-absorption in near band gap exciton emitters is discussed elaborately by Yan et al.^149^

Plastic scintillators show very promising values for their pulse quality and pulse duration. However, one of the main problems with these materials is their low density, typically about 1 g/cm^3^, and low stopping power. Plastic scintillators mostly contain low-Z elements like hydrogen, carbon, nitrogen, and oxygen. Because their exact composition is not always known, it was not possible to calculate the value of ρZ_*eff*_^3.5^ for these materials. The low stopping power and effective atomic number does not only result in the need for very large pixels to absorb the energy of incident X-ray photons but also makes Compton scattering the dominant interaction mechanism. Scattered X-ray photons can be detected in neighboring pixels, resulting in multiple detected events for one incident X-ray photon.

**Figure 4:**
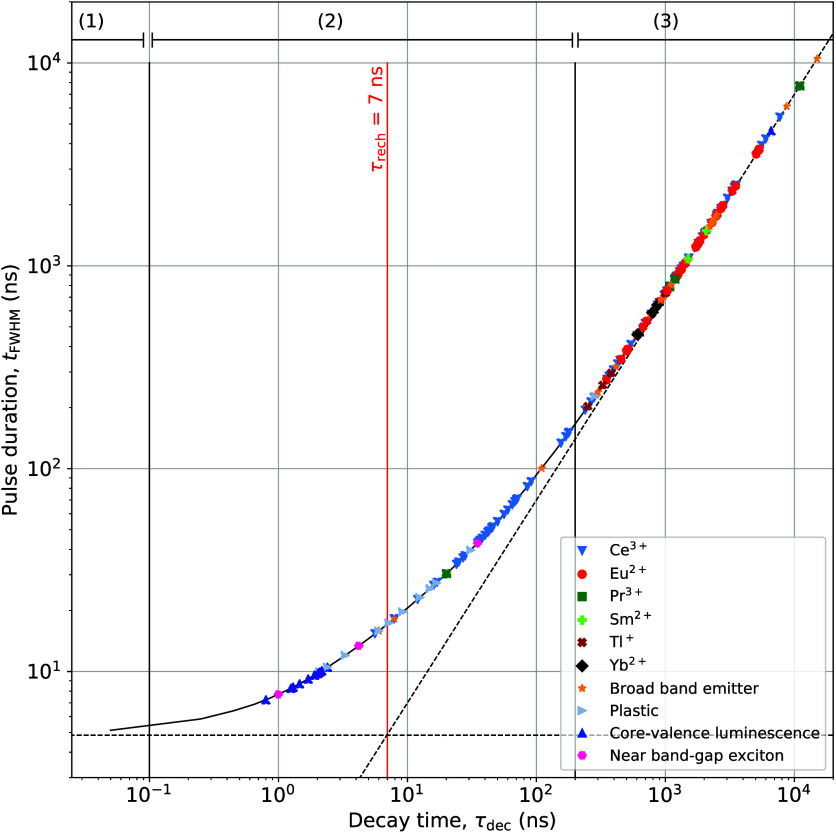
Scintillation decay time (τ_dec_) versus calculated pulse duration (*t*_fwhm_) for Ce^3+^-, Pr^3+^-, Eu^2+^-, Yb^2+^-, Sm^2+^ codoped, and Tl^+^-doped scintillators, intrinsic scintillators showing core–valence luminescence, broad-band emission, near band gap exciton emission, and plastic scintillators. The red vertical line indicates the recharge time of the SiPM. The numbers (1), (2), and (3) indicate the regimes where *t*_fwhm_(τ_rech_), *t*_fwhm_(τ_rech_,τ_dec_), and *t*_fwhm_(τ_dec_). The two black vertical lines deliniate the different regimes.

Figure 4 shows the decay time versus the pulse duration for the collected data points. The red vertical line indicates the 7 ns recharge time of the SiPM used to calculate the pulse duration. Three different regimes can be identified in this plot. When the decay time is significantly shorter than the SiPM recharge time, the pulse duration is determined almost entirely by the recharge time (*t*_fwhm_(τ_rech_)) and, therefore, the pulse duration is of a given, finite length, no matter how fast the scintillator emits. This regime lies on the left-hand side of the left-most black vertical line in Figure 4. There are no data points in this regime. On the other hand, when the decay time is significantly longer than the recharge time, the pulse duration is mainly determined by the decay time (*t*_fwhm_(τ_dec_)). This regime lies on the right-hand side of the rightmost black vertical line. In these extreme cases, the pulse duration can be estimated based on a single exponential decay, as indicated by the horizontal and diagonal dashed asymptotes, respectively. In the middle regime (*t*_fwhm_(τ_dec_, τ_rech_)), where τ_rech_ and τ_dec_ are of comparable magnitudes, the pulse duration is determined by both of them. This is the region of Figure 4 in which interesting data points are found.

## VI Scintillator Engineering

In addition to selecting scintillators for application in an indirect PCD it is also possible to optimize one. There are various ways in which this can be achieved, for example T_50_ engineering or codoping. More recently it has also been suggested that the properties of hybrid organic–inorganic perovskites can be tailored by specifically changing the dielectric constant of the organic layer.^125,126^

### VI.A T_50_ Engineering

The performance of a scintillator in an indirect PCD can sometimes be improved by quenching its emission, thereby making its decay faster. This strategy shortens the pulse duration and allows the scintillator to handle a higher count rate. Even though quenching causes a decrease in light yield, from a theoretical perspective, the amplitude of the scintillation pulse is independent of temperature everywhere along the quenching curve. The same applies to the pulse quality, as long as the decay time is much longer than the recharge time of the SiPM (in other words, in the regime in which the pulse duration is mainly determined by τ_dec_). For illustrative purposes, the effect on the decay time and pulse quality of quenching a scintillator’s emission is modeled assuming a hypothetical scintillator with a light yield of 140,000 ph/MeV and a decay time τ_dec_ = 140 ns at zero kelvin. It is assumed that the quenching curve can be described using a single-barrier Arrhenius equation:^154,155^
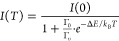
18
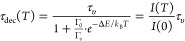
19where *I*(*T*) represents the luminescence intensity at the absolute temperature *T*, *I*(0) the luminescence intensity at *T* = 0 K, Γ_0_ the attempt rate for thermal quenching, Γ_υ_ the radiative decay rate, *k*_B_ the Boltzmann constant, and Δ*E* the energy barrier. In eq 19, τ_dec_(*T*) represents the decay time at the absolute temperature *T* and τ_υ_ is the decay time at *T* = 0.

Based on eqs 18 and 19, the change in light yield and decay time can be calculated along the quenching curve; when the light yield is halved, the decay time is also halved. This allows for the calculation of multiple points along the quenching curve. Based on the resulting values of the light yield and decay time, the pulse duration and pulse quality are calculated. Additionally, the light yield and decay time are used as input for the experimentally validated model of van der Sar et al. to simulate detector output pulses.^22−25^

**Figure 5:**
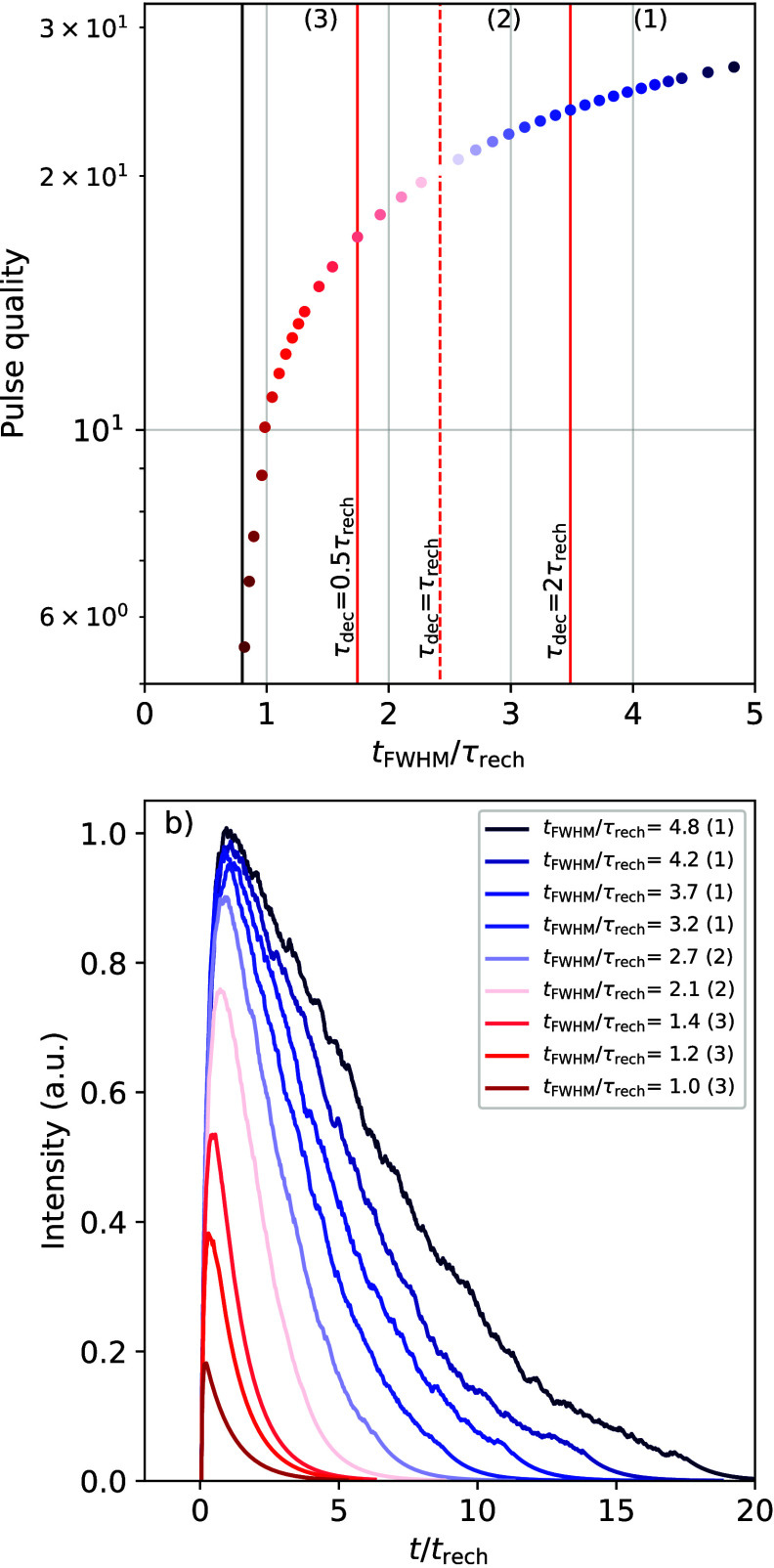
(a) *t*_fwhm_/τ_rech_ versus the pulse quality, determined from simulated pulses, for different points along the quenching curve of a scintillator. (b) Selection of the simulated detector output pulses along the curve shown in (a). The numbers (1), (2), and (3) indicate the regimes in which τ_dec_ ≫ τ_rech_, τ_dec_ ≈ τ_rech_, and τ_dec_ ≪ τ_rech_, respectively.

Figure 5a shows the calculated variation of the pulse quality along the quenching curve. The pulse duration plotted on the *X*-axis is normalized by dividing it by the recharge time of the SiPM. A selection of simulated detector output pulses is shown in Figure 5b. The data points in 5a and the simulated pulses in Figure 5b can be grouped into three different regimes, based on the recharge and decay time. In regime (1), τ_dec_ ≫ τ_rech_. Quenching of the decay time in this regime results in a decrease of the pulse duration with almost no change of the pulse amplitude and therefore the pulse quality. In regime (2), τ_dec_ ≈ τ_rech_. Quenching of the decay time in this regime results in a decrease of the pulse duration together with a moderate decrease in the amplitude of the simulated pulses and, therefore, the pulse quality. In regime (3), τ_dec_ ≪ τ_rech_. Quenching the decay time in this regime results in almost no decrease of the pulse duration but leads to a significant decrease of the pulse amplitude and, therefore, the pulse quality. In Figure 5a, this can be observed by the curve reaching a vertical asymptote along which only the pulse quality decreases. This behavior can also be observed in the simulated detector output pulses in Figure 5b. Ideally, a scintillator should thus be quenched from the first to the second regime, since further quenching of the decay time to the third regime will mostly result in a decrease of the total pulse intensity with almost no improvement of the pulse duration.

The single-barrier Arrhenius equation, assumed above, shows that there are two options to quench a scintillator, either by increasing the temperature (T) or by decreasing the energy barrier (Δ*E*). Given the requirement of room temperature operation, the only viable option is to decrease the energy barrier, i.e., decreasing the quenching temperature T_50_, which represents the temperature at which the quenching curve reaches 50% of its maximum intensity.

The energy barrier, or T_50_, can be changed via material engineering; this can also be referred to as T_50_-engineering. An example of this can be found in the family of Ce^3+^-doped garnets. Depending on the cation composition, thermal quenching can either take place through thermal ionization to the conduction band or interconfigurational crossover from the 5d excited state to the 4f ground state.^156,157^ The activation energy of both processes strongly depends on composition and T_50_ values can be gradually tuned down from approximately 650 K in Y_3_Al_5_O_12_:Ce^3+^ to well below room temperature in Y_3_Ga_5_O_12_:Ce^3+^.^158^ By replacing Al^3+^ with Ga^3+^, the room temperature decay time could be shortened from approximately 80 to 18.6 ns.^159^

This is only one example of a system for which the quenching temperature can be engineered. As shown in Figure 3, there are a number of Ce^3+^-doped materials with pulse durations approaching that of CdTe that could be improved and tailored via this approach. This can, however, also be used for intrinsic broad-band emitting scintillators. One of the main question of T_50_-engineering is how the nonproportionality of the scintillator will be influenced. In principle, changes in composition should lead to minimal changes of the nonproportionality, but this needs to be confirmed experimentally.

### VI.B Co-doping

A more commonly used strategy to engineer or fine-tune the properties of a scintillator is to add additional dopants, also referred to as codoping.^160,161^ Even high-quality scintillation crystals contain several different types of lattice defects, i.e., point defects, interstitials, dislocations, etc. The formation of such defects cannot completely be suppressed by optimizing the synthesis procedure. However, the influence of these defects can be mitigated via the addition of a specific codopant. This approach has for example been used to improve the nonproportionality of LaBr_3_:Ce^3+^ via codoping with Sr^2+^,^26,49,162^ or SrI_2_:Eu^2+^ and KCaI_3_:Eu^2+^ by codoping with Zr^4+^.^163,164^ Co-doping can also be used to reduce afterglow, for example in Gd_2_O_2_S:Pr^3+^^165^ and CsI:Tl^+^.^166−171^ Other interesting examples are the improvement of the temporal response of the lutetium- and yttrium-based orthosilicates ((Lu,Y)_2_SiO_5_:Ce^3+^) codoped with Ca^2+^^51,172−174^ and the garnets ((Gd,Lu,Y)_3_(Al,Ga)_5_O_12_:Ce^3+^) codoped with Mg^2+^.^175−179^

It should be noted that the influence of a codopant is very hard to predict and that codopants can have multiple effects at the same time. A good example of this is LaBr_3_:Ce^3+^,Sr^2+^. On the one hand, codoping with Sr^2+^ improves the light yield and nonproportionality. On the other hand, it introduces multiple slower decay components, which is detrimental for application in an indirect PCD. The codoping approach thus requires a careful evaluation of the effects on all material properties.

### VI.C Dielectric Engineering

The hybrid organic–inorganic perovskites and perovskite-related compounds are an emerging group of intrinsic scintillators. These compounds can be engineered in many different ways, for example by doping the inorganic layer or by using a mix of organic molecules.^180−189^ Another approach to engineer these materials is by changing the dielectric constant of the organic layer. This approach has been explored by Xia et al., who replaced phenethylammonium (PEA) on the A site of PEA_2_PbBr_4_ with benzimidazole (BM).^125^ This approach is referred to as dielectric engineering.^125,126^ The dielectric constant of BM is smaller than that of PEA; this enhances the exciton confinement by increasing the exciton binding energy and decreasing the exciton lifetime. Xia et al. report a scintillation decay time of 3.22 ns for PEA(_2_PbBr_4_, which decreases to 0.97 ns for BM_2_PbBr_4_. It should be noted that both of these compounds are still near-bandgap excitonic emitters, which means that self-absorption would still pose a challenge. Nonetheless, this approach may be very interesting for future developments in this class of hybrid organic–inorganic materials.

## VII Conclusions

The suitability of different types of scintillators for application in indirect PCDs has been assessed using three figures of merit: pulse intensity, pulse duration, and pulse quality. Based on these figures of merit, which are based on emissive properties, it is concluded that Ce^3+^- or Pr^3+^-doped materials form interesting classes of scintillators. Other groups that look promising are near band gap exciton emitters, plastics, and core–valence emitters. The application of these materials, however, is hampered by secondary problems or challenges, e.g., the match between the emission wavelength and PDE of the SiPM, presence of slow decay components, density, or nonproportionality. Examples are given of how scintillators can be engineered to optimize their emissive characteristics for use in indirect PCDs. If T50 engineering is used, the decay time of the scintillator should be quenched to values approximately similar to the recharge time of the SiPM. Further quenching will lead to a decrease in the pulse intensity without any gain in pulse duration. This illustrates how choosing and/or engineering a suitable material for an indirect PCD depends on not only the material properties; the properties of the SiPM are just as important.

## Supporting Information Available

Decay times and amplitudes of scintillators for which an average decay time is tabulated (PDF)
