# Supplementary material for: Guidelines for the Selection of Scintillators for Indirect Photon-Counting X-ray Detectors
Source: Chem Mater. 2025 Feb 26;37(5):1716–40. doi: 10.1021/acs.chemmater.4c03437 (PMC11905209; doi:10.1021/acs.chemmater.4c03437)
Supplement: Supplementary file 1 — cm4c03437_si_001.pdf [file cm4c03437_si_001.pdf]

Guidelines for the Selection of Scintillators for Indirect Photon-Counting X-ray Detectors Supplementary Information

J. Jasper van Blaaderen[1], Casper van Aarle[1], David Leibold[1], Pieter Dorenbos[1], Dennis R. Schaart[1,2]

1: Delft University of Technology,

Faculty of Applied Sciences,

Department of Radiation Science and Technology,

Mekelweg 15, 2629 JB Delft, Netherlands

2: Holland Proton Therapy Center,

Huismansingel 4,

2629 JH Delft, The Netherlands

TABLE I: Components of the decay time of Ce^{3+} for which average values are tabulated in the main text.

Compound	τ_{dec} (ns)	Ref
$LaCl_3$	26 (70%)<br>210 (30%)	[1–3]
$LuCl_3$	56 (45%)<br>337 (25%)<br>5900 (30%)	[2, 4]
$Cs_2LiGdCl_6$	129 (51%)<br>573 (32%)<br>8900 (17%)	[5]
$Cs_2LiLaCl_6$	2 (9%)<br>40 (11%)<br>450 (80%)	[6]
KGd_2Cl_7	50 (40%)<br>200 (60%)	[7]
$Rb_2LiGdCl_6$	107 (21%)<br>791 (41%)<br>4100 (38%)	[8]
$Cs_2NaLaCl_6$	40 (33%)<br>105 (33%)<br>338 (14%)<br>1848 (20%)	[9]
$Cs_2NaGdCl_6$	107 (23%)<br>697 (41%)<br>3600 (36%)	[10, 11]

Cs_3LaCl_6	79 (32%)<br>291 (33%)<br>1243 (35%)	[9]
Cs_3GdCl_6	39 (7%)<br>129 (12%)<br>462 (15%)<br>2110 (66%)	[10]
$Tl_2LiGdCl_6$	34 (81%)<br>191 (10%)<br>1200 (9%)	[12]
Tl_2LiYCl_6	57 (3%)<br>431 (64%)<br>1055 (33%)	[13]
Tl_2LaCl_5	36 (90%)<br>217 (6%)<br>1500 (34%)	[14, 15]
$Tl_2LiLuCl_6$	72 (8%)<br>366 (30%)<br>1500 (62%)	[16]
Tl_2GdCl_5	32 (76%)<br>271 (10%)<br>1600 (14%)	[17]
$Cs_2NaCeCl_6$	91 (36%)<br>601 (33%)<br>3200 (31%)	[18]
$Cs_2LiCeCl_6$	101 (42%)<br>557 (35%)<br>2900 (23%)	[19]
Cs_3CeCl_6	50 (50%)<br>300 (50%)	[20]

$Rb_2LiCeCl_6$	71 (85%)<br>405 (15%)	[21]
$Cs_2NaLaCl_6$	66 (26%)<br>340 (18%)<br>1620 (56%)	[9]
$CsSrCl_3$	3.8 (23%)<br>57 (23%)<br>626 (54%)	[22]
$Cs_2LiLaBr_6$	122 (61%)<br>661 (39%)	[23–25]
$LaBr_3$:Ce,Sr	16.8 (56%)<br>56 (16%)<br>240 (16%)<br>1530 (12%)	[26–28]
$LuBr_3$	19 (33%)<br>369 (22%)<br>3600 (45%)	[2, 4]
$GdBr_3$	20 (26%)<br>212 (65%)<br>1300 (9%)	[29]
Cs_2LiYBr_6	65 (11%)<br>2500 (89%)	[30, 31]
$Cs_2NaLaBr_6$	48 (20%)<br>205 (21%)<br>854 (23%)<br>4021 (36%)	[9, 32]
Cs_2 $NaLuBr_6$	61 (65%)<br>350 (35%)	[32]

Cs_2NaYBr_6	61 (26%)<br>350 (23%)<br>2700 (51%)	[32]
Cs_3LaBr_6	44 (17%)<br>124 (18%)<br>399 (20%)<br>2230 (45%)	[9, 33]
Rb_2LiYBr_6	42 (35%)<br>250 (32%)<br>1400 (33%)	[34]
$Rb_2LiLaBr_6$	26 (5%)<br>310 (37%)<br>1100 (58%)	[35]
$BaBr_2$	80 (27%)<br>490 (22%)<br>2100 (51%)	[36]
$Cs_2NaGdBr_6$	72 (67%)<br>266 (22%)<br>698 (11%)	[10, 37]
$Cs_2NaLaBr_3I_3$	78 (25%)<br>294 (40%)<br>1338 (34%)	[38]
$Cs_2NaYBr_3I_3$	56 (47%)<br>284 (25%)<br>2060 (28%)	[38]
Cs_3GdBr_6	72 (26%)<br>270 (25%)<br>1421 (49%)	[10]

$Cs_2LiGdBr_6$	73 (63%)<br>542 (14%)<br>3900 (23%)	[39]
$Tl_2LiGdBr_6$	29 (92%)<br>197 (8%)	[40]
$Cs_2NaCeBr_6$	140 (94%)<br>880 (6%)	[41]
$Rb_2LiCeBr_6$	55 (87%)<br>284 (13%)	[5]
$CsCe_2Br_7$	78 (52%)<br>316 (32%)<br>1723 (16%)	[42]
$CeBr_3$:Sr	23 (97%)<br>211 (3%)	[43–45]
$Cs_2LiCeBr_6$	86 (76%)<br>444 (8%)<br>3800 (16%)	[46]
$LaBr_{2.25}I_{0.75}$	31 (70%)<br>224 (30%)	[47]
LuI_3	23 (75%)<br>120 (25%)	[48–50]
$Cs_3Lu_2I_9$	18 (2%)<br>42 (6%)<br>120 (40%)<br>510 (52%)	[33]
SrI_2:Ce,Na	32 (46%)<br>450 (54%)	[51]
$YAlO_3$	26 (90%)<br>67 (10%)	[52–54]

$LuAlO_3$	17 (90%)<br>88 (10%)	[55]
Gd_2SiO_5	56 (89%)<br>600 (11%)	[56, 57]
$K_2Lu(PO_4)_2$	34 (81%)<br>1200 (19%)	[58]

TABLE II: Components of the decay time of Eu^{2+} for which average values are tabulated in the main text.

Compound	τ_{dec} (ns)	Ref
$BaCl_2$	25 (15%)<br>138 (21%)<br>642 (64%)	[36, 59]
$BaBr_2$	35 (8%)<br>415 (47%)<br>814 (45%)	[36, 60]
BaBrI	297 (23%)<br>482 (77%)	[61, 62]
KSr_2Br5	520 (21%)<br>1076 (79%)	[63]
Rb_4CaBr_6	2830 (79%)<br>8520 (21%)	[64]
$TlSr_2I_5$	525 (73%)<br>3300 (27%)	[65]
KSr_2I_5	990 (89%)<br>5000 (11%)	[66]
$KCaI_3$:Eu,Zr	1030 (88%)<br>2260 (12%)	[67]

TABLE III: Components of the decay time of Pr^{3+} for which average values are tabulated in the main text.

Compound	τ_{dec} (ns)	Ref
$(Lu,Y)_3Al_5O_{12}$:Pr	43.9 (55%)<br>333 (24%)<br>1374 (21%)	[68–70]
$(Lu,Y)_3Al_5O_{12}$:Pr,Li	47.8 (47%)<br>374 (27%)<br>1461 (26%)	[70]
$CsBa_2I_5$:Eu,Sm	240 (6%)<br>2090 (94%)	[71]
NaI:Tl,Ca	172 (92%)<br>860 (8%)	[72]
NaI:Tl,Sr	173 (94%)<br>830 (6%)	[72]

TABLE IV: Components of the decay time of intrinsic scintillators for which average values are tabulated in the main text.

Compound	τ_{dec} (ns)	Ref
$TlMgCl_3$	46 (9%)<br>166 (23%)<br>449 (68%)	[73]
$TlCaCl_3$	317 (44%)<br>727 (56%)	[74]
Tl_2HfCl_6	300 (15%)<br>1100 (85%)	[75, 76]
Tl_2ZrCl_6	500 (2%)<br>2300 (98%)	[75, 76]
$TlSr_2Br_5$	390 (66%)<br>1900 (34%)	[77]
$TlCaBr_3$	56 (76%)<br>2490 (24%)	[78]
$TlCaI_3$	62 (13%)<br>200 (62%)<br>1440 (25%)	[73]
$TlSr_2I_5$	151 (33%)<br>605 (39%)<br>3000 (28%)	[65]
$RbSrI_3$	375 (71%)<br>1300 (29%)	[79]
$CaWO_4$	1400 (30%)<br>9200 (70%)	[80–82]
$CdWO_4$	5000 (60%)<br>20000 (40%)	[83, 84]

$PbWO_4$	2.2 (50%%)<br>9.9 (34%)<br>39 (16%)	[85–87]
Sc_2O_3	64 (8%)<br>295 (92%)	[88]

TABLE V: Components of the decay time of intrinsic scintilators which show core-valence emission for which average values are tabulated in the main text.

Compound	τ_{dec} (ns)	Ref
BaF_2	1 (20%)<br>630 (80%)	[89–91]
Cs_2LiYCl_6	4 (10%)<br>6600 (90%)	[30, 92]

TABLE VI: Components of the decay time of plastic scintilators for which average values are tabulated in the main text.

Compound	τ_{dec} (ns)	Ref
p-Terphenyl	2.1 (97%)<br>22.6 (3%)	[93, 94]

[1] E. V. D. van Loef, P. Dorenbos, C. W. E. van Eijk, K. Kramer, H. U. Gudel, Applied Physics Letters 77 (2000) 10, https://doi.org/10.1063/1.1308053

[2] O. Guillot-Noel, J. T. M. De Haas, P. Dorenbos, C. W. E. van Eijk, K. Kramer, H. U. Gundel, Journal of Luminescence 85 (1999) 1-3, https://doi.org/10.1016/S0022-2313(99)00063-0

[3] K. S. Shah, J. Glodo, M. Klugerman, L. Cirignano, W. W. Moses, S. E. Derenzo, M. J. Weber, Nuclear Instrumetns and Methods in Physics Research Section A: Accelerators, Spectrometers, Detectors and Associated Equipment 505 (2003) 1-2, https://doi.org/10.1016/S0168-9002(03)01024-6

[4] E. V. D. van Loef, P. Dorenbos, C. W. E. van Eijk, K. W. Kramer, H. U. Gudel, Nuclear Instrumetns and Methods in Physics Research Section A: Accelerators, Spectrometers, Detectors and Associated Equipment 496 (2003) 1, https://doi.org/10.1016/S0168-9002(02)01634-0

[5] G. Rooh, H. J. kim, S. Kim, Radiation Measurements 45 (2010) 3-6, https://doi.org/10.1016/j.radmeas.2009.10.018

[6] J. Glodo, R. Hawrami, E. van Loef, W. Higgins, U. Shirwadkar, K. S. Shah, Proceedings Volume 7449, Hard X-ray, Gamma-ray, and neutron detector physuics XI: 74490E (2009), https://doi.org/10.1117/12.830127

[7] M. Zhuravleva, K. Yang, A. Green, C. L. Melcher, Journal of Crystal Growth 318 (2011) 1, https://doi.org/10.1016/j.jcrysgro.2010.10.206

[8] G. Rooh, H. J. Kim, H. Park, S. Kim, Journal of Crystal Growth 377 (2013) 28-31, https://doi.org/10.1016/j.jcrysgro.2013.04.036

[9] G. Gundiah, K. Brennan, Z. Yan, E. C. Samulon, G. Wu, G. A. Bizarri, S. E. Derenzo, E. D. Bourret-Courchesne, Journal of Luminescence 149 (2014) 374-384, https://doi.org/10.1016/j.jlumin.2013.09.057

[10] E. C. Samulon, G. Gundiah, M. Gascon, I. V. Khodyuk, S. E. Derenzo, G. A. Bizarri, E. D. Bourret-Courchesne, Journal of Luminescence 153 (2014) 64-72, https://doi.org/10.1016/j.jlumin.2014.02.021

[11] G. Rooh, H. J. Kim, H. Park, S. Kim, H. Jiang, IEEE Transactions on Nuclear Science 61 (2014) 1, https://doi.org/10.1109/TNS.2013.2283882

[12] H. J. Kim, G. Rooh, H. Park, S. Kim, Journal of Luminescence 164 (2015) 86-89.

https://doi.org/10.1016/j.jlumin.2015.03.026

[13] R. Hawrami, E. Ariesanti, L. Soundara-Pandian, J. Glodo, K. S. Shah, IEEE Transactions on Nuclear Science 63 (2016) 6, https://doi.org/10.1109/TNS.2016.2627523

[14] H. J. Kim, G. Rooh, S. Kim, Journal of Luminescence 186 (2017) 219-222, https://doi.org/10.1016/j.jlumin.2017.02.042

[15] R. Hawrami, E. Ariesanti, H. Wei, J. Finkelstein, J. Glodo, K. S. Shah, Nuclear Instruments and Methods in Physics Research Section A: Accelerators, Spectrometers, Detectors and Associated Equipment 869 (2017) 107-109, https://doi.org/10.1016/j.nima.2017.06.016

[16] G. Rooh, H. J. Kim, J. Jang, S. Kim, Journal of Luminescence 187 (2017) 347-351, https://doi.org/10.1016/j.jlumin.2017.03.051

[17] A. Khan, G. Rooh, H. J. Kim, S. Kim, Journal of Alloys and Compounds 741 (2018) 878-882, https://doi.org/10.1016/j.jallcom.2018.01.204

[18] G. Rooh, H. Kang, H. J. Kim, H. Park, S. Kim, Journal of Crystal Growth 311 (2009) 8, https://doi.org/10.1016/j.jcrysgro.2009.01.091

[19] G. Rooh, H. J. Kim, S. Kim, IEEE Transactions on Nuclear Science 57 (2010) 3, https://doi.org/10.1109/TNS.2009.2037903

[20] M. Zhuravleva, K. yang, C. L. Melcher, Journal of Crystal Growth 318 (2011) 1, https://doi.org/10.1016/j.jcrysgro.2010.11.090

[21] G. Rooh, H. J. Kim, H. Park, S, Kim, IEEE Transactions on Nuclear Science 59 (2012) 5, https://doi.org/10.1109/TNS.2012.2200907

[22] Y. Fujimoto, K. Saeki, D. Nakauchi, T. Yanagida, M. Koshimizu, K. Asai, Sensors and Materials 29 (2017) 10, https://doi.org/10.18494/SAM.2017.1622

[23] J. Glodo, E. van Loef, R. Hawrami, W. M. Higgins, A. Churilov, U. Shirwadkar, K. S. Shah, IEEE Transactions on Nuclear Science 58 (2011) 1, https://doi.org/10.1109/TNS.2010.2098045

[24] J. Qin, J. Xiao, T. Zhu, X. Lu, Z. Han, M. Wang, L. Jiang, Y. Mou, J. Sun, Z. Wen, X. Wang, Nuclear Instruments and Methods in Physics Research Section A: Accelerators, Spectrometers, Detectors and Associated Equipment 905 (2018) 112-118, https://doi.org/10.1016/j.nima.2018.05.006

[25] K. Yang, P. R. Menge, J. Lejay, V. Ouspenski, IEEE Nuclear Science Symposium and Medical Imaging Conference (2013) 1-6, https://doi.org/10.1109/NSSMIC.2013.6829676

[26] M. S. Alekhin, J. T. M. de Haas, I. V. Khodyuk, K. W. Kramer, P. R. Menge, V. Ouspenski, P. Dorenbos, Applied Physics Letters 102 (2013) 161915, https://doi.org/10.1063/1.4803440

[27] K. Yang, P. R. Menge, J. J. Buzniak, V, ouspenski, IEEE Nuclear Science Symposium and Medical Imaging Conference Record (NSS/MIC) 2012 308-311, https://doi.org/10.1109/NSSMIC.2012.6551113

[28] M. S. Alekhin, D. A. Biner, K. W. Kramer, P. Dorenbos, Journal of Applied Physics 113 (2013) 224904, https://doi.org/10.1063/1.4810848

[29] E. V. D. van Loef, P. Dorenbos, C. W. E. van Eijk, K. W. Kramer, H. U. Gudel, Optics Communications 189 (2001) 4-6, https://doi.org/10.1016/S0030-4018(01)01039-2

[30] A. Bessiere, P. Dorenbos, C. W. E. van Eijk, K. W. Kramer, H. U. Gudel, IEEE Transactions on Nuclear Science 51 (2004) 5, https://doi.org/10.1109/TNS.2004.834957

[31] U. Shirwadkar, J. Glodo, E. van Loef, R. hawrami, S. Mukhopadhyay, K. S. Shah, IEEE Nuclear Science Symposium and Medical Imaging Conference 2010 1585-1588, https://doi.org/10.1109/NSSMIC.2010.5874043

[32] M. D. Birowosuto, P. Dorenbos, C. W. E. van Eijk, K. W. Kramer, H. U. Gudel, Journal of Physics: Condensed Matter 18 (2006) 6133, https://doi.org/10.1088/0953-8984/18/26/031

[33] M. D. Birowosuto, P. Dorenbos, C. W. E. van Eijk, K. W. Kramer, H. U. Gudel, Physica Statutus Solidi A Applications and Materials Science 204 (2007) 3, https://doi.org/10.1002/pssa.200622459

[34] M. D. Birowosuto, P. Dorenbos, J. T. M. de Haas, C. W. E. van Eijk, K. W. Kramer, H. U. Gudel, Journal of Applied Physics 101 (2007) 6, https://doi.org/10.1063/1.2713948

[35] M. D. Birowosuto, P. Dorenbos, J. T. M. de Haas, C. W. E. van Eijk, K. W. Kramer, H. U. Gudel, IEEE Transactions on Nuclear Science 55 (2008) 3, https://doi.org/10.1109/TNS.2008.922826

[36] J. Selling, S. Schweizer, M. D. Birowosuto, P. DOrenbos, IEEE Transactions on Nuclear Science 55 (2008) 3, https://doi.org/10.1109/TNS.2008.922825

[37] G. Rooh, H. J. Kim, H. Park, S. Kim, Journal of Luminescence 132 (2012) 3, https://doi.org/10.1016/j.jlumin.2011.10.015

[38] H. Wei, L. Stand, M. Zhuravleva, F. Meng, V. Martin, C. L. Melcher, Optical Materials 38 (2014) 154-160, https://doi.org/10.1016/j.optmat.2014.09.038

[39] G. Rooh, H. J. Kim, H. Park, S. Kim, Journal of Luminescence 146 (2014) 404-407,

https://doi.org/10.1016/j.jlumin.2013.09.047

[40] H. J. Kim, G. Rooh, H. Park, S. Kim, Radiation Measurements, 90 (2016) 279-281, https://doi.org/10.1016/j.radmeas.2015.12.021

[41] S. Kim, G. Rooh, H. J. Kim, W. Kim, U. hong, IEEE Transactions on Nuclear Science 57 (2010) 3, https://doi.org/10.1109/TNS.2010.2041789

[42] Y. Wu, H. Shi, B. C. Chakoumakos, M. Zhuravleva, M.-H. Du, C. L. Melcher, Journal of Materials Chemistry C 3 (2015) 11366-11376, https://doi.org/10.1039/C5TC02721G

[43] P. Guss, M. E. Foster, B. M. Wong, F. P. Doty, K. Shah, M. R. Squillante, U. Shirwadkar, R. Hawrami, J. Tower, D. Yuan, Journal of Applied Physics 115 (2014) 034908, https://doi.org/10.1063/1.4861647

[44] F. G. A. Quarati, M. S. Alekhin, K. W. Kramer, P. Dorenbos, Nuclear Instruments and Methods in Physics Research Section A: Accelerators, Spectrometers, Detectors and Associated Equipment 735 (2014) 655-658, https://doi.org/10.1016/j.nima.2013.10.004

[45] R. H. P. Awater, K. W. Kramer, P. Dorenbos, IEEE Transactions on Nuclear Science 62 (2015) 5, https://doi.org/10.1109/TNS.2015.2463736

[46] J. K. Cheon, S. Kim, G. Rooh, J.H. So, H. J. Kim, H. Park, Nuclear Instruments and Methods in Physics Research Section A: Accelerators, Spectrometers, Detectors and Associated Equipment 652 (2011) 1, https://doi.org/10.1016/j.nima.2011.02.038

[47] M. D. Birowosuto, P. Dorenbos, K. W. Kramer, H. U. Gudel, Journal of Applied Physics 103 (2008) 103517, https://doi.org/10.1063/1.2930884

[48] Birowosuto, P. Dorenbos, C. W. E. van Eijk, K. W. Kramer, H. U. Gudel, IEEE Transactions on Nuclear Science 52 (2005) 4, https://doi.org/10.1109/TNS.2005.852630

[49] M. D. Birowosuto, P. Dorenbos, C. W. E. van Eijk, K. W. Kramer, H. U. Gudel, Journal of Applied Physics 99 (2005) 1235200, https://doi.org/10.1063/1.2207689

[50] K. S. Shah, J. Glodo, M. Klugerman, W. Higgins, T. Gupta, P. Wong, W. W. Moses, S. E. Derenzo, M. J. Weber, P. Dorenbos, IEEE Transactgions on Nuclear Science 51 (2004) 5, https://doi.org/10.1109/TNS.2004.832321

[51] C. M. Wilson, E. V. van Loef, J. Glodo, N. Cherepy, G. Hull, S. Payne, W.-S. Choong, W. Moses, K. S. Shah, Proceedings Volume 7079, Hard X-ray, Gamma-Ray and Neutron Detector PhysicsX, 707917 (2008), https://doi.org/10.1117/12.806291

[52] J. T. M. de Haas, P. Dorenbos, C. W. E. van Eijk, Nuclear Instruments and Methods in Physics

Research Section A: Accelerators, Spectrometers, Detectors and Associated Equipment 537 (2005) 1-2, https://doi.org/10.1016/j.nima.2004.07.243

[53] M. Kapusta, M. Balcerzyk, M. Moszynski, J. Pawelke, Nuclear Instruments and Methods in Physics Research Section A: Accelerators, Spectrometers, Detectors and Associated Equipment 421 (1999) 3, https://doi.org/10.1016/S0168-9002(98)01232-7

[54] S. E. Dorenzo, M. J. Weber, W. W. Moses, C. Dujardin, IEEE Transactions on Nuclear Science 47 (2000) 3, https://doi.org/10.1109/23.856531

[55] M. Moszynski, D. Wolski, T. Ludziejewski, M. Kapusta, A. Lempicki, C. Brecher, D. Wisniewski, A. J. Wojtowicz, Nuclear Instruments and Methods in Physics Research Section A: Accelerators, Spectrometers, Detectors and Associated Equipment 385 (1997) 1, https://doi.org/10.1016/S0168-9002(96)00875-3

[56] E. Sakai, IEEE Transactions on Nuclear Science 34 (1987) 1, https://doi.org/10.1109/TNS.1987.4337375

[57] M. Balcerzyk, M. Moszynski, M. Kapusta, D. Wolski, J. Pawelke, C. L. Melcher, IEEE Transactions on Nuclear Science 47 (2000) 4, https://doi.org/10.1109/23.872971

[58] D. Wisniewski, A. J. Wojtowicz, W. Drozdowski, J. M. Farmer, L. A. Boatner, Journal of Alloys and Compounds 380 (2004) 1-2, https://doi.org/10.1016/j.jallcom.2004.03.042

[59] Z. Yan, G. Bizarri, E. Gourret-Courchesne, Nuclear Instruments and Methods in Physics Research Section A: Accelerators, Spectrometers, Detectors and Associated Equipment 698 (2013) 7-10 https://doi.org/10.1016/j.nima.2012.09.026

[60] Z. Yan, G. Gundiah, G. A. Bizarri, E. C. Samulon, S. E. Derenzo, E. D. Bourret-Courchesne, Nuclear Instruments and Methods in Physics Research Section A: Accelerators, Spectrometers, Detectors and Associated Equipment 735 (2014) 83-87, https://doi.org/10.1016/j.nima.2013.09.021

[61] G. Bizarri, E. D. Bourret-Courchesne, Z. Yan, S. E. Derenzo, IEEE Transactions on Nuclear Science 58 (2011) 6, https://doi.org/10.1109/TNS.2011.2166999

[62] E. D. Bourret-Courchesne, G. Bizarri, S. M. Hanrahan, G. Gundiah, Z. Yan, S. E. Derenzo, Nuclear Instruments and Methods in Physics Research Section A: Accelerators, Spectrometers, Detectors and Associated Equipment 613 (2010) 1, https://doi.org/10.1016/j.nima.2009.11.036

[63] L. Stand, M. Zhuravleva, H. Wei, C. L. Melcher, Optical Materials 46 (2015) 59-63,

https://doi.org/10.1016/j.optmat.2015.04.002

[64] K. S. Pestovich, L. Stand, E. van Loef, C. L. Melcher, M. Zhuravleva, IEEE Transactions on Nuclear Science 70 (2023) 7, https://doi.org/10.1109/TNS.2023.3280733

[65] H. J. Kim, G. Rooh, A. Khan, H. Park, S. Kim, Optical materials 82 (2018) 7-10, https://doi.org/10.1016/j.optmat.2018.05.036

[66] L. Stand, M. Zhuravleva, A. Lindsey, C. L. Melcher, Nuclear Instruments and Methods in Physics Research Section A: Accelerators, Spectrometers, Detectors and Associated Equipment 780 (2015) 40-44, https://doi.org/10.1016/j.nima.2015.01.052

[67] Y. Wu, Q. Li, D. J. rutstrom, M. Zhuravleva, M. Loyd, l. Stand, M. Koschan, C. L. Melcher, Physica Status Solidi Rapid Research Letters 12 (2018) 2, https://doi.org/10.1002/pssr.201700403

[68] J. A. Mares, M. Nikl, A. Beitlerova, P. Horodysky, K. Blazek, K. Bartos, C. D'Ambrosio, IEEE Transactions on Nuclear Science 59 (2012) 5, https://doi.org/10.1109/TNS.2012.2191573

[69] W. Drozdowski, K. Brylew, A. J. Wojtowicz, J. Kisielewski, M. Swirkowicz, T. Lukasiewicz, J. T. M. de Haas, P. Dorenbos, Optical Materials Express 4 (2014) 6, https://doi.org/10.1364/OME.4.001207

[70] C. Foster, Y. Wu, M. Koschan, C. L. Melcher, Physica Status Solidi Rapid Research Letters 12 (2018) 9, https://doi.org/10.1002/pssr.201800280

[71] W. Wolszczak, K. W. Kramer, P. Dorenbos, Physica Status Solidi Rapid Research Letters 13 (2019) 9, https://doi.org/10.1002/pssr.201900158

[72] K. Yang, P. R. Menge, Journal of Applied Physics 118 (2015) 21, https://doi.org/10.1063/1.4937126

[73] R. Hawrami, E. Ariesanti, H. Wei, J. Finkelstein, J. Glodo, K. S. Shah, Journal of Crystal Growth 475 (2017) 216-219, https://doi.org/10.1016/j.jcrysgro.2017.06.012

[74] A. Khan, G. Rooh, H. J. Kim, H. Park, S. Kim, Radiation Measurements 107 (2017) 115-118, https://doi.org/10.1016/j.radmeas.2017.09.003

[75] R. Hawrami, E. Ariesanti, V. Buliga, A. Burger, S. Lam, S. Motakef, Journal of Crystal Growth 531 (2020) 125316, https://doi.org/10.1016/j.jcrysgro.2019.125316

[76] Y. Fujimoto, K. Saeki, D. Nakauchi, T. Yanagida, M. Koshimizu, K. Asai, Sensors and Materials 30 (2018) 7, https://doi.org/10.18494/SAM.2018.1927

[77] G. Rooh, A. Khan, H. J. Kim, H. Park, S. Kim, Optical Materials 73 (2017) 523-526,

https://doi.org/10.1016/j.optmat.2017.08.047

[78] E. van Loef, L. S. Pandian, N. Kaneshige, G. Ciampi, L. Stand, D. Rutstrom, Y. Tratsiak, M. Zhuravleva, C. Melcher, K. S. Shah, IEEE Transactions on Nuclear Science 70 (2023) 7, https://doi.org/10.1109/TNS.2023.3258065

[79] K. S. Pestovich, L. Stand, C. L. Melcher, E. van Loef, M. Zhuravleva, Journal of Crystal Growth 627 (2024) 1, https://doi.org/10.1016/j.jcrysgro.2023.127540

[80] V. B. Mikhailik, H. Kraus, S. Henry, A. J. B. Tolhurst, Physical Review B 75 (2007) 184308, https://doi.org/10.1103/PhysRevB.75.184308

[81] Y. G. Zdesenko, F. T. Avigone III. V. B. Brudanin, F. Danevich, S. S. Nagorny, I. M. Solsky, V. I Tretyak, Nuclear Instruments and Methods in Physics Research Section A: Accelerators, Spectrometers, Detectors and Associated Equipment 538 (2005) 1-3, https://doi.org/10.1016/j.nima.2004.09.030

[82] M. Moszynski, M. Balcerzyk, W. Czarnacki, A. Nassalski, T. Szcesniak, H. Kraus, V. B. Mikhailik, I. M. Solskii, Nuclear Instruments and Methods in Physics Research Section A: Accelerators, Spectrometers, Detectors and Associated Equipment 553 (2005) 3, https://doi.org/10.1016/j.nima.2005.07.052

[83] C. L. Melcher, R. A. Manente, J. S. Schweitzer, IEEE Transactions on Nclear Science 36 (1989) 1, https://doi.org/10.1109/23.34629

[84] I. Holl, E. Lorenz, G. Mageras, IEEE Transactions on Nuclear Science 35 (1998) 1, https://doi.org/10.1109/23.12684

[85] A. A. Annenkov, M. V. Korzhik, P. Lecoq, Nuclear Instruments and Methods in Physics Research Section A: Accelerators, Spectrometers, Detectors and Associated Equipment 490 (2002) 1-2, https://doi.org/10.1016/S0168-9002(02)00916-6

[86] M. Kobayashi, M. Ishii, Y. Usuki, H. Yahagi, Nuclear Instruments and Methods in Physics Research Section A: Accelerators, Spectrometers, Detectors and Associated Equipment 333 (1993) 2-3, https://doi.org/10.1016/0168-9002(93)91187-R

[87] P. Lecoq, I. Dafinei, E. Auffray, M. Schneegans, M. V. Korlzhik, O. V. Missevitch, V. B. Pavlenko, A. A. Fedorov, A. N. Annenkov, V. L. Kostylev, V. D. Ligun, Nuclear Instruments and Methods in Physics Research Section A: Accelerators, Spectrometers, Detectors and Associated Equipment 365 (1995) 2-3, https://doi.org/10.1016/0168-9002(95)00589-7

[88] A. Fukabori, L. An, A. Ito, V. Chani, K. Kamada, T. Goto, A. Yoshikawa, IEEE Transactions

on Nuclear Science 59 (2012) 5, https://doi.org/10.1109/TNS.2012.2206211

[89] M. Laval, M. Moszynski, R. Allemand, E. Cormoreche, P. Guinet, R. Odru, J. Vacher, Nuclear Instruments and Methods in physics Reserach 206 (1983) 1-2, https://doi.org/10.1016/0167-5087(83)91254-1

[90] M. Biasini, D. B. Cassidy, S. H. M. Dena, H. K. M. Tanaka, A. P. Mills, Nuclear Instruments and Methods in Physics Research Section A: Accelerators, Spectrometers, Detectors and Associated Equipment 553 (2005) 3, https://doi.org/10.1016/j.nima.2005.07.022

[91] V. Nanal, B. B. Back, D. J. Horman, Nuclear Instruments and Methods in Physics Research Section A: Accelerators, Spectrometers, Detectors and Associated Equipment 389 (1997) 3, https://doi.org/10.1016/S0168-9002(97)00326-4

[92] C. M. Combes, P. Dorenbos, C. W. E. van Eijk, K. W. Kramer, H. U. Gudel, Journal of Luminescence 82 (1999) 4, https://doi.org/10.1016/S0022-2313(99)00047-2

[93] T. Yanagida, K. Watanabe, Y. Fujimoto, Nuclear Instruments and Methods in Physics Research Section A: Accelerators, Spectrometers, Detectors and Associated Equipment 784 (2015) 111-114, https://doi.org/10.1016/j.nima.2014.12.031

[94] M. De Gerone, M. Biasotti, V. Ceriale, D. Corsini, F. Gatti, A. Orlando, G. Pizzigoni, Nuclear Instruments and Methods in Physics Research Section A: Accelerators, Spectrometers, Detectors and Associated Equipment 824 (2016) 192-193, https://doi.org/10.1016/j.nima.2015.11.021
